# Pain Management During Neonatal Blood Sampling: An Overview of Systematic Reviews

**DOI:** 10.1002/pne2.70044

**Published:** 2026-09-08

**Authors:** Libby G. Lord, Jane E. Harding, Caroline A. Crowther, Luling Lin

**Affiliations:** ^1^ Liggins Institute, University of Auckland Auckland New Zealand

**Keywords:** breast feeding, infant, kangaroo‐mother care method, newborn, pain, prevention and control, sucrose

## Abstract

This overview of systematic reviews aimed to identify effective pain management strategies for neonatal blood sampling. Searches were conducted in four databases from 2014 to April 2026. Eligible studies were systematic reviews including randomized or quasi‐randomized controlled trials assessing pain management in neonates during blood sampling. Reviews were required to have searched at least two databases within the past 10 years, reported their search strategy, and conducted a risk‐of‐bias assessment. The search identified 2463 records; 22 reviews (13 Cochrane and two non‐Cochrane pairwise reviews, and seven network meta‐analyses) met inclusion criteria. Interventions evaluated included opioids, paracetamol, topical anesthetics, breastfeeding or breastmilk, sucrose, music medicine, acupuncture, and other non‐pharmacological methods. Sucrose with or without non‐nutritive sucking was effective in reducing pain scores during heel pricks in preterm and term neonates (mean difference −1.74, 95% confidence interval [−2.11, −1.37], moderate‐certainty). Skin‐to‐skin contact also reduced pain scores compared with control (mean difference −3.47 [−5.55, −1.38], moderate‐certainty). Recent network meta‐analyses generally supported parent‐led interventions and oral sweet solutions as the most effective strategies. Breastfeeding, supplemental breastmilk, acupuncture, and light reduction were promising but require further evidence. For other interventions, evidence was insufficient to draw conclusions. Evidence from systematic reviews and network meta‐analyses indicates that sucrose and skin‐to‐skin contact effectively reduce procedural pain in neonates. Parent‐led and other non‐pharmacological interventions, including breastfeeding, also appear beneficial and should increasingly be considered part of family‐centred neonatal pain care where feasible.

## 1 Introduction

Neonatal hypoglycaemia is a common condition affecting up to 15% of newborns [1]. Severe or symptomatic episodes have been associated with poorer neurodevelopmental outcomes in later childhood [2, 3]. Standard clinical practice is to monitor blood glucose concentrations in at‐risk neonates to determine treatment needs [4]. Blood samples for this purpose are usually collected by heel prick [4].

Heel pricks are painful [5, 6] and pain may be associated with adverse neurodevelopment in preterm neonates [7]. Further, neonates at risk of hypoglycaemia typically undergo multiple tests. One study reported medians of 9 (range 1–22) tests in at‐risk infants [8], and another reported means of 7.8 in hypoglycaemic infants and 3.8 in those who were not [9].

As painful blood sampling procedures are currently unavoidable, strategies to minimize neonatal pain are essential. Increasing emphasis has been placed on parent‐led and other non‐pharmacological approaches that support the biopsychosocial and emotional wellbeing of both infants and families [10]. However, the implementation of these interventions may be influenced by healthcare system, organizational, and clinical practice factors [11]. This overview therefore reviewed systematic reviews of pain management for neonates during blood sampling to identify effective strategies.

Since neonates are non‐verbal, pain must be assessed using physiological or behavioral indicators [12]. Validated scales include the Premature Neonate Pain Profile (PIPP) [13, 14], the Neonatal Pain Scale (NIPS) [15], the Douleur Aiguë Nouveau‐né (DAN) scale [16], the Neonatal Facial Coding System (NFCS) [17] and the Neonatal Pain, Agitation and Sedation Scale (N‐PASS) [18].

Pain may be measured during the procedure (reactivity) or after it (response), or as a physiological change from baseline. Indicators include heart rate, respiratory rate, oxygen saturation, blood pressure, cerebral oxygenation (near‐infrared spectroscopy), brain activity (electroencephalography), and hormonal responses such as salivary cortisol [19, 20].

## 2 Methods

This overview of systematic reviews followed Chapter V of the Cochrane Handbook for Systematic Reviews of Interventions [21] and was registered prospectively on PROSPERO (CRD42023474078). The protocol is available in Appendix S1.

We searched Ovid MEDLINE, Ovid Embase, CINAHL Complete and the Cochrane Database of Systematic Reviews from inception to 27 August 2025 and updated on 20 April 2026 (Appendix S2 for search strategies). Eligible systematic reviews included only evidence from randomized controlled trials (RCTs) and/or quasi‐RCTs, reported their search strategy, searched at least two databases, assessed risk‐of‐bias, and conducted searches after 2013. No language restrictions were applied. Both Cochrane and non‐Cochrane reviews were included if they involved neonates (up to 28 days of age or study defined) undergoing blood sampling, with any pain‐reducing intervention (pharmacological or non‐pharmacological) compared with standard care, placebo, or no intervention. The primary outcome was validated composite pain scores (Appendix S3) during or after the procedure. Secondary outcomes included physiological measures, adverse effects, parent or clinician satisfaction, and cost of the intervention (review‐defined). Network meta‐analyses (NMAs) were additionally considered because of their increasing prominence in neonatal pain research and their ability to provide comparative estimates across multiple interventions. Although NMAs may include intervention‐versus‐intervention comparisons beyond the original scope of direct intervention‐versus‐control comparisons, they were included if they incorporated at least one comparison between an intervention and standard care, placebo, or no intervention in neonatal procedural pain populations. Given the substantial overlap in primary trials between the pairwise systematic reviews and NMAs, NMA findings were interpreted narratively as complementary comparative evidence providing treatment rankings.

Two reviewers (LL and LGL) independently screened titles, abstracts, and full‐text articles in Covidence [22]. Overlapping reviews were resolved by prioritizing Cochrane reviews for methodological rigor. For skin‐to‐skin contact, the 2017 Cochrane review was supplemented with newer trials from the recent search. Data were extracted independently into prespecified forms: one for systematic reviews and another for additional primary trials. Extracted data included outcomes, search methods, interventions, analyses, subgroups, risk of bias, GRADE assessment, funding, inclusion criteria, number and location of studies, and procedure types. Where meta‐analysis was absent, narrative synthesis was used. Certainty of evidence was assessed using GRADE [23], extracted from the reviews or reassessed when not reported. We also reviewed the study authors' interpretation of the magnitude of effect, using recommendations from the Cochrane Handbook [24].

Methodological quality of the systematic reviews was appraised independently by two reviewers using AMSTAR2 [25], assessing domains including research question clarity, protocol specification, duplicate processes, search strategy, study description, risk‐of‐bias handling, funding reporting, synthesis methods, and conflicts of interest. Discrepancies were resolved through discussion. Methodological quality was not formally assessed for the included NMAs because existing appraisal tools may not adequately capture methodological considerations specific to NMAs. Instead, characteristics of the included NMAs, including network characteristics, risk of bias assessments, and certainty of evidence assessments, were described narratively.

Meta‐analysis was performed in RevMan 5.4 using a random‐effects model. Mean differences (MDs) or standardized mean differences (SMDs) with 95% confidence intervals (CIs) were reported for continuous outcomes, and relative risks (RRs) for dichotomous outcomes. A *p* value < 0.05 was considered statistically significant. Narrative summaries were provided where meta‐analysis was not feasible.

## 3 Results

**FIGURE 1:**
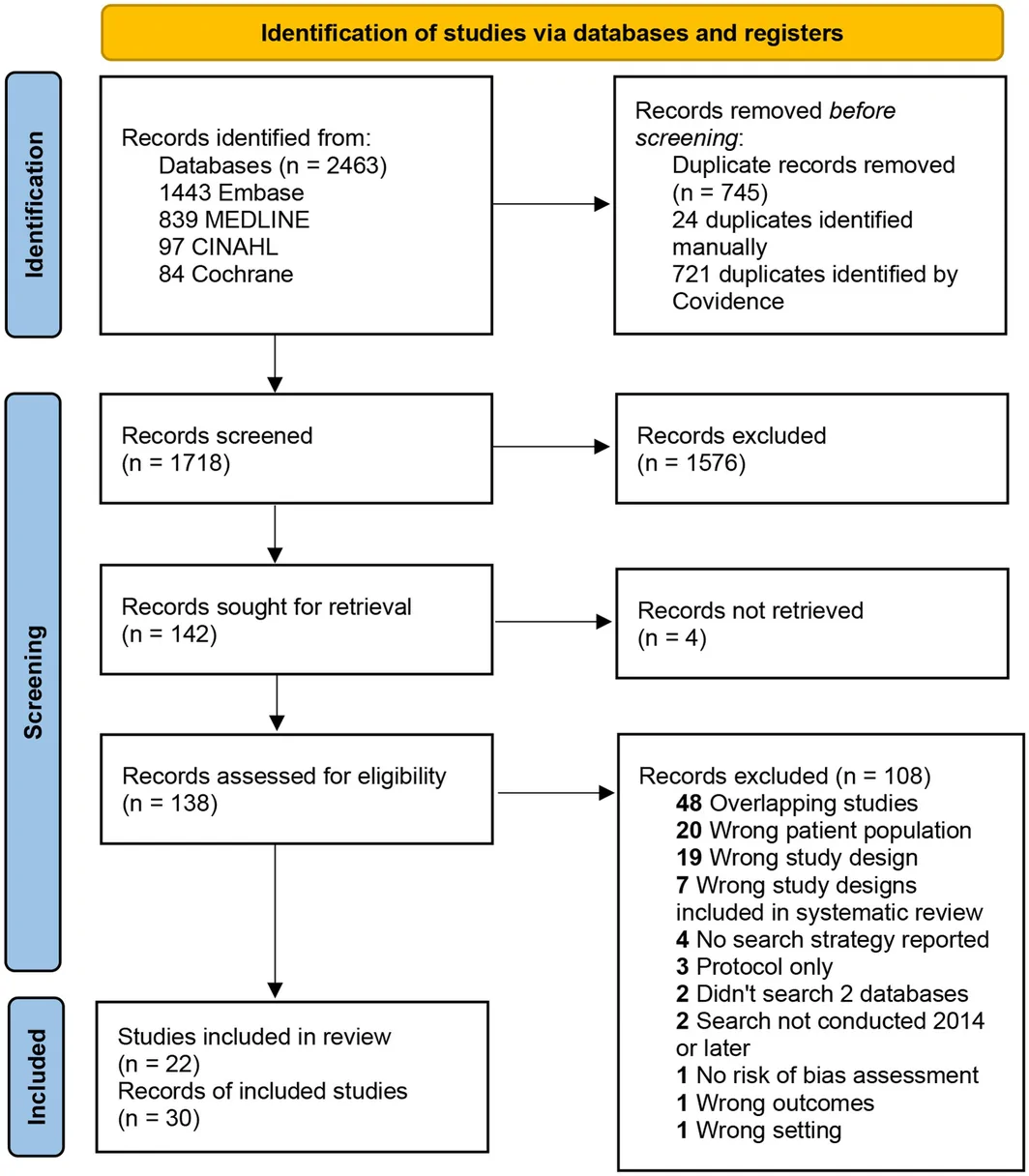
PRISMA flow diagram of selected systematic reviews.

The search identified 2463 records. After removing duplicates, 1718 titles/abstracts and 142 full texts were screened. Twenty‐two reviews met the inclusion criteria including 13 Cochrane [26, 27, 28, 29, 30, 31, 32, 33, 34, 35, 36, 37] and two non‐Cochrane [38, 39] pairwise reviews, and seven NMAs [40, 41, 42, 43, 44, 45, 46] (Figure 1), that included 8–105 RCTs with 506–7049 participants. Interventions assessed included opioids, paracetamol, topical anesthetics, breastfeeding or breast milk, sucrose, music, acupuncture, and other non‐pharmacological approaches (Table 1 for pairwise reviews and Appendix S1 for NMAs). Only skin‐to‐skin contact had sufficient data for meta‐analysis beyond the 2017 Cochrane review.

**TABLE 1:** Characteristics of included reviews.

Author/year	Intervention	Date of search	Number of included trials (design, publication years and procedures)	Number of participants in included trials	Study risk of bias assessments	AMSTAR 2 rating
Bertol 2025	Human milk odor	July 2025	8 RCTs	451	Bias from randomization process: 8 RCT low, 0 RCT some concern, 0 RCT high risk Bias due to deviations from intended interventions: 6 RCT low, 2 RCT unclear risk, 0 RCT high risk Bias due to missing outcome data: 8 RCT low, 0 RCT some concern, 0 RCT high risk Bias in measurement of the outcomes: 6 RCT low, 0 RCT some concern, 2 RCT high risk Bias in selection of the reported result: 8 RCT low, 0 RCT some concern, 0 RCT high risk Overall risk of bias: 6 RCT low, 0 RCT some concern, 2 RCT high risk	Moderate
Bueno 2026	Sucrose	July 2025	29 RCTs (all are venepuncture, including cross‐over and cluster RCTs)	2764	Sequence generation: 29 RCT low, 0 RCT unclear risk, 0 RCT high risk Allocation concealment: 15 RCT low risk, 14 RCT unclear risk, 0 RCT high risk Blinding (participants and personnel): 12 RCT low risk, 15 RCT unclear risk, 2 RCT high risk Blinding (outcome assessors): 24 RCT low risk, 4 RCT unclear risk, 1 RCT high risk Incomplete outcome data: 29 RCT low, 0 RCT unclear risk, 0 RCT high risk Selective reporting: 7 RCT low risk, 22 RCT unclear risk, 0 RCT high risk Other: 22 RCT low risk, 3 RCT unclear risk, 2 RCT high risk Overall: Not reported	High
Cabano 2025	Acupuncture	August 2023	11 RCTs (only 7 compare acupuncture to placebo or no treatment so are eligible for our review, these all focus on heel lance)	521	Sequence generation: 4 RCT low risk, 4 RCT unclear risk, 0 RCT high risk Allocation concealment: 7 RCT low risk, 1 RCT unclear risk, 0 RCT high risk Blinding (participants and personnel): 4 RCT low risk, 4 RCT unclear risk, 0 RCT high risk Blinding (outcome assessors): 5 RCT low risk, 3 RCT unclear risk, 0 RCT high risk Incomplete outcome data: 6 RCT low risk, 1 RCT unclear risk, 1 RCT high risk Selective reporting: 2 RCT low risk, 6 RCT unclear risk, 0 RCT high risk Other: 7 RCT low risk, 0 RCT unclear risk, 1 RCT high risk Overall: Not reported	High
Costa 2022	Music‐based interventions	July 2021	39 RCTs (only 10 involving blood sampling) 2003–2021 7 heel prick 2 venipuncture 1 “blood sampling”	595	Joanna Briggs Institute Critical Appraisal Checklist for Randomized Controlled Trials was used rather than Cochrane Risk of Bias, further breakdown of different aspects of the checklist can be found in the supplement of Costa et al. [39]. Overall: 8 RCT low risk, 1 RCT moderate risk, 1 RCT high risk	Critically low
Foster 2017	Topical anesthesia	May 2016	8 RCTs 1995–2008 3 heel prick 3 venipuncture 2 other (lumbar puncture, injection)	506	Sequence generation: 4 RCT low risk, 4 RCT unclear risk, 0 RCT high risk Allocation concealment: 8 RCT low risk, 0 RCT unclear risk, 0 RCT high risk Blinding (participants and personnel): 7 RCT low risk, 0 RCT unclear risk, 1 RCT high risk Blinding (outcome assessors): 5 RCT low risk, 2 RCT unclear risk, 0 RCT high risk, 1 RCT not assessed Incomplete outcome data: 6 RCT low risk, 2 RCT unclear risk, 0 RCT high risk Selective reporting: 1 RCT low risk, 7 RCT unclear risk, 0 RCT high risk Other: 7 RCT low risk, 0 RCT unclear risk, 1 RCT high risk Overall: Not reported	Moderate
Johnston 2017	Skin‐to‐skin contact	February 2017	25 RCTs and quasi‐RCTs 2000–2015 19 heel prick 1 heel prick and venipuncture 5 other (injection, “vaccination”, tape removal)	2001	Sequence generation: 14 RCT low risk, 10 RCT unclear risk, 1 RCT high risk Allocation concealment: 9 RCT low risk, 15 RCT unclear risk, 1 RCT high risk Blinding (participants and personnel, outcome assessors): 6 RCT low risk, 12 RCT unclear risk, 7 RCT high risk Incomplete outcome data: 18 RCT low risk, 6 RCT unclear risk, 1 RCT high risk Selective reporting: 21 RCT low risk, 2 RCT unclear risk, 2 RCT high risk Other: 13 RCT low risk, 7 RCT unclear risk, 4 RCT high risk 1 RCT not reported Overall: Not reported	Moderate
Kinoshita 2023	Opioids (primarily morphine, fentanyl and remifentanil)	December 2021	13 RCTs and quasi‐RCTs 1991–2021 2 heel prick 1 heel prick and ROP exam 10 other (ROP exam or laser treatment, central catheter placement, routine care procedures and tracheal suction, lumbar puncture)	823	Sequence generation: 8 studies low risk, 5 studies unclear risk, 0 studies high risk Allocation concealment: 11 studies low risk, 2 studies unclear risk, 0 studies high risk Blinding (participants and personnel): 7 studies low risk, 2 studies unclear risk, 4 studies high risk Blinding (outcome assessors): 11 study low risk, 2 studies unclear risk, 0 studies high risk Incomplete outcome data: 12 studies low risk, 1 study unclear risk, 0 studies high risk Selective reporting: 8 studies low risk, 5 studies unclear risk, 0 studies high risk Other: 10 studies low risk, 3 studies unclear risk, 0 studies high risk Overall: Not reported	High
Ohlsson 2020	Paracetamol (acetaminophen)	May 2016	9 RCTs 5 heel prick 1998–2009 4 other (Eye examination)	728	Sequence generation: 9 RCT low risk Allocation concealment: 8 RCT low risk, 1 RCT unclear risk Blinding (participants and personnel): 8 RCT low risk, 1 RCT unclear risk Blinding (outcome assessors): 9 RCT low risk Incomplete outcome data: 9 RCT low risk, 1 RCT high risk Selective reporting: 2 RCT low risk, 7 RCT unclear risk Other: 9 RCT low risk Overall: 9 RCT low	High
Pillai Riddell 2023	Nonpharmacological, excluding kangaroo care, sucrose, breastfeeding/breast milk, music.	July 2022	105 RCTs conducted in neonates (preterm or full term) 1982–2015 63 heel prick 15 venipuncture 3 unspecified blood sampling procedure 25 other (vaccine/vitamin needle, diaper change, endotracheal suctioning, weighing, ROP screening, peripheral cannulation, orogastric tube insertion)		Sequence generation: 80 RCT low risk, 19 RCT unclear risk, 6 RCT high risk Allocation concealment: 30 RCT low risk, 56 RCT unclear risk, 19 RCT high risk Blinding (participants and personnel): 18 RCT low risk, 28 RCT unclear risk, 59 RCT high risk Blinding (outcome assessors): 29 RCT low risk, 32 RCT unclear risk, 44 RCT high risk Incomplete outcome data: 78 RCT low risk, 16 RCT unclear risk, 11 RCT high risk Selective reporting: 26 RCT low risk, 79 RCT unclear risk, 0 RCT high risk Other: 98 RCT low risk, 3 RCT unclear risk, 4 RCT high risk Overall: 1 RCT low risk, 25 RCT unclear risk, 79 RCT high risk	High
Shah 2023	Breastfeeding or breast milk	August 2022	66 RCT 1997–2022 39 heel prick 11 venipuncture 16 other (vaccination, ROP exam, suctioning, tape removal)	> 6200	Sequence generation: 40 RCT low risk, 24 RCT unclear risk, 2 RCT high risk Allocation concealment: 36 RCT low risk, 21 RCT unclear risk, 9 RCT high risk Blinding (participants and personnel): 21 RCT low risk, 24 RCT unclear risk, 21 RCT high risk Blinding (outcome assessors): 33 RCT low risk, 14 RCT unclear risk, 19 RCT high risk Incomplete outcome data: 59 RCT low risk, 2 RCT unclear risk, 5 RCT high risk Selective reporting: 61 RCT low risk, 1 RCT unclear risk, 4 RCT high risk Other: 2 RCT low risk, 64 RCT unclear risk, 0 RCT high risk Overall: Not reported	High
Stevens 2016	Sucrose	February 2016	74 RCTs 1993–2016 38 heel prick 9 venipuncture 1 heel prick and venipuncture 1 arterial puncture 25 other (injection, bladder catheterisation, tube insertion, ROP exam, circumcision, multiple painful procedures) Extracted the 2 venipuncture studies with eligible outcomes	7049	Sequence generation: 2 RCT low risk, 0 RCT unclear risk, 0 RCT high risk Allocation concealment: 2 RCT low risk, 0 RCT unclear risk, 0 RCT high risk Blinding (participants and personnel): 1 RCT low risk, 1 RCT unclear, 0 RCT high risk Blinding (outcome assessors): 1 RCT low risk, 1 RCT unclear, 0 RCT high risk Incomplete outcome data: 2 RCT low risk, 0 RCT unclear risk, 0 RCT high risk Selective reporting: 0 RCT low risk, 2 RCT unclear risk, 0 RCT high risk Other: 2 RCT low risk, 0 RCT unclear risk, 0 RCT high risk Overall: Not reported	High
Yamada 2023	Sucrose for heel prick	February 2022	55 RCT 1995–2021 51 heel prick 1 heel prick and venipuncture 4 multiple painful procedures	6273	Sequence generation: 35 RCT low risk, 20 RCT unclear risk, 0 RCT high risk Allocation concealment: 24 RCT low risk, 18 RCT unclear risk, 13 RCT high risk Blinding (participants and personnel): 25 RCT low risk, 10 RCT unclear risk, 20 RCT high risk Blinding (outcome assessors): 25 RCT low risk, 10 RCT unclear risk, 20 RCT high risk Incomplete outcome data: 44 RCT low risk, 7 RCT unclear risk, 4 RCT high risk Selective reporting: 13 RCT low risk, 41 RCT unclear risk, 1 RCT high risk Other: 53 RCT low risk, 2 RCT unclear risk, 0 RCT high risk	High

Abbreviations: RCT, randomized controlled trial; ROP, retinopathy of prematurity.

Based on AMSTAR2, eight reviews were high quality [26, 27, 30, 31, 32, 33, 34, 35], two moderate [28, 29], one low [38] and one critically low [39] (Table 2). Two reviews could not be assessed due to lack of relevant or usable data [36, 37].

**TABLE 2:** Results of assessment of the methodological quality of systematic reviews using AMSTAR2.

Review ID	Research questions and inclusion criteria include the components of PICO?	Review methods established prior to conduct of review and any significant protocol deviations reported?	Explanation of selection of study designs for inclusion?	Comprehensive literature search strategy?	Study selection performed in duplicate?	Data extraction performed in duplicate?	List of excluded studies provided, and inclusions justified?	Description of included studies in adequate detail	Satisfactory technique for assessing risk‐of‐bias of included studies?	Reported on sources of funding for included studies?	If meta‐analysis performed, appropriate methods used for statistical combination of results?	If meta‐analysis performed, was potential impact of RoB in individual studies on results of meta‐analysis/other evidence synthesis assessed?	Accounted for RoB in individual studies when interpreting/discussing results of review?	Provided satisfactory explanation and discussion of heterogeneity in results	If meta‐analysis performed, was adequate investigation into publication bias carried out and likely impact on results discussed?	Potential conflicts of interest of review authors reported (including funding)?	Overall
Bertol 2026	Yes	Yes	Yes	Partial Yes	Yes	Yes	No	Yes	Yes	No	Yes	No	No	Yes	Yes	Yes	Low
Bueno 2026	Yes	Yes	Yes	Yes	Yes	Yes	Yes	Yes	Yes	Yes	Yes	Yes	Yes	Yes	Yes	Yes	High
Cabano 2025	Yes	Yes	Yes	Yes	Yes	Yes	Yes	Yes	Yes	Yes	Yes	Yes	Yes	Yes	Yes	Yes	High
Costa 2022	Yes	No	Yes	Partial Yes	Yes	No	No	Partial yes	Partial yes	No	N/A	N/A	No	No	N/A	Yes	Critically low
Foster 2017	Yes	Yes	Yes	Yes	Yes	No	Yes	Yes	Yes	No	Yes	Yes	Yes	Yes	Yes	Yes	Moderate
Johnston 2017	Yes	Yes	Yes	Partial Yes	Yes	Yes	Yes	Yes	Yes	No	Yes	Yes	Yes	Yes	Yes	Yes	Moderate
Kinoshita 2023	Yes	Yes	Yes	Yes	Yes	Yes	Yes	Yes	Yes	Yes	Yes	Yes	Yes	Yes	Yes	Yes	High
Ohlsson 2020	Yes	Yes	Yes	Partial Yes	Yes	Yes	Yes	Yes	Yes	No	Yes	Yes	Yes	Yes	Yes	Yes	High
Pillai Riddell 2023	Yes	Yes	Yes	Partial Yes	Yes	Yes	Yes	Yes	Yes	Yes	Yes	Yes	Yes	Yes	Yes	Yes	High
Shah 2023	Yes	Yes	Yes	Yes	Yes	Yes	Yes	Yes	Yes	Yes	Yes	Yes	Yes	Yes	Yes	Yes	High
Stevens 2016	Yes	Yes	Yes	Yes	No	Yes	Yes	Yes	Yes	Yes	Yes	Yes	Yes	Yes	Yes	Yes	High
Yamada 2023	Yes	Yes	Yes	Partial Yes	Yes	Yes	Yes	Yes	Yes	Yes	Yes	Yes	Yes	Yes	Yes	Yes	High

Abbreviation: RoB, risk of bias.

The NMAs incorporated both direct and indirect comparisons using Bayesian or frequentist approaches, with treatment ranking methods such as SUCRA or P‐scores. Risk of bias assessments were conducted using the Cochrane Risk of Bias tool. However, six of the seven NMAs applied ROB 2 at the study level by assigning overall study judgments, rather than conducting outcome‐specific assessments as recommended by ROB 2 guidance [47]. Most reviews reported predominantly low risk or some concerns, although several identified substantial proportions of studies at high risk of bias. Certainty of evidence assessments were variably undertaken using GRADE for NMA or CINeMA frameworks, with certainty ranging from high to very low across interventions and outcomes, while three NMAs did not formally assess certainty of evidence. In general, evidence certainty was downgraded because of imprecision, inconsistency, heterogeneity, incoherence, and within‐study bias, with only a limited number of interventions supported by moderate‐ or high‐certainty evidence (Appendix S4).

### 3.1 Effective Interventions

#### 3.1.1 Sucrose

##### 3.1.1.1 Primary Outcome

###### 3.1.1.1.1 Heel Prick [35]

*During Procedure:* No data reported.

*After Procedure:* In preterm and term neonates, sucrose with or without nonnutritive sucking likely reduced PIPP scores at 30 s (MD −1.74, 95% CI [−2.11, −1.37], moderate‐certainty) and 60 s (MD –2.14 [−3.34, −0.94], moderate‐certainty) compared to control. In subgroup analysis, this effect was seen for preterm infants at 30 s (MD –1.88 [−2.32, −1.44], low‐certainty), but there was little to no effect in term infants at 30 s (MD −0.87 [−1.8, 0.06], low‐certainty).

The evidence is very uncertain about the effect of sucrose with or without nonnutritive sucking on DAN scores at 30 s after heel lancing in term neonates (MD –1.90 [−8.58, 4.78]).

Compared to water, sucrose with or without nonnutritive sucking likely reduced NIPS score immediately after heel prick in term neonates (MD −2.00 [−2.42, −1.58], moderate‐certainty).

The earlier Cochrane review by Stevens et al. [34] included heel prick studies prior to the updated review by Yamada et al. [35], with narrative reporting from five additional studies comparing sucrose to control. All reported reduced pain with sucrose: one showed lower NIPS scores 1 min after the procedure in preterm neonates, three reported reduced behavioral scores (facial expressions and presence of cry), and one found reduced mean PIPP scores.

###### 3.1.1.1.2 Venipuncture [26]

*During Procedure:* In both preterm and term neonates, sucrose (with or without nonnutritive sucking) probably reduces pain intensity scores during and shortly after venepuncture (SMD −0.82 [−1.02, −0.63], moderate‐certainty).

*After Procedure:* Sucrose with nonnutritive sucking resulted in lower pain intensity scores 1 min after venepuncture (MD −9.15, [−9.91, −8.39], high‐certainty). Sucrose with or without nonnutritive sucking resulted in lower pain intensity scores (measured by NIPS and PIPP) at 1 min after venepuncture in two studies. The evidence is uncertain about the effect of sucrose (20%–24%) without nonnutritive sucking on pain intensity scores 2 min after venepuncture (SMD −0.99, [−1.31, −0.68], very low‐certainty).

##### 3.1.1.2 Secondary Outcomes

###### 3.1.1.2.1 Heel Prick [35]

Evidence suggests that sucrose compared to control results in little to no difference in heart rate (MD 2.21 bpm [−3.95, 8.37]), respiratory rate (MD –1.00 rpm [−7.64, 5.64]), oxygen saturation (MD –5.00% [−12.79, 2.79]), or nociceptive‐specific brain activity (mean weight by EEG MD 0.02 [−0.05, 0.09]) during heel prick. Evidence is very uncertain regarding the effect of sucrose compared to control on the percentage change in heart rate 1 min after lancing (MD 0.90 [−5.81, 7.61]).

Compared to the control group, evidence is very uncertain about the effect of sucrose on total crying time after lancing in term infants (MD –30.74 s [−40.73, −20.74]). Sucrose may reduce the time from the start of vocalization until a 5 s quiet interval compared to control (MD –53.18 [−76.90, −39.46], low‐certainty). Evidence is very uncertain about the effect of sucrose compared to control on the time from the start of vocalization until before the first inspiration (MD –5.00 [−17.40, −7.40]). The Stevens et al. [34] review also reported narrative findings from six additional studies, all indicating improved behavioral outcomes in those receiving sucrose compared to water or routine care (e.g., shorter cry duration, reduced facial action and increased quiet sleep).

Adverse effects were reported in ten studies in the Yamada et al. [35] systematic review, with no difference between sucrose and placebo groups. Those reported included choking, oxygen desaturation, spitting up, vomiting, and abdominal distension, but all resolved spontaneously. One study in the Yamada et al. [35] systematic review reported no difference in blood glucose concentrations between sucrose and water groups. Review authors concluded that adverse events were rare and minor.

###### 3.1.1.2.2 Venipuncture [34]

Two studies suggested that sucrose compared to control may result in little to no effect on the duration of crying (24%–30% sucrose, MD –16.50 [−71.41, 38.41], 50% sucrose, MD –14.00 [−51.79, 23.79]) with very uncertain evidence.

#### 3.1.2 Skin‐To‐Skin

Where not otherwise referenced, data are from the Cochrane systematic review of skin‐to‐skin contact for pain management for neonates during painful procedures [29]. The characteristics of individual trials were presented in Appendix S5.

##### 3.1.2.1 Primary Outcome: Composite Pain Scores

*During the Procedure:* Wang [48] found that skin‐to‐skin contact reduced PIPP during heel prick compared to control (MD −2.82 [−3.38, −2.26], high‐certainty evidence).

In the Johnston et al. [29] Cochrane review, one RCT reported reduced pain during heel prick in the skin‐to‐skin group compared to control, but raw data were not provided [29]. Two RCTs not included in the Cochrane review [49] were meta‐analyzed and the results showed that skin‐to‐skin compared to control likely reduces NFCS during heel prick (MD −0.89 [−1.16, −0.61]), moderate‐certainty evidence (Appendix S6).

*After the Procedure:* The Johnston et al. [29] Cochrane review, updated with findings from Shukla [50] reported that skin‐to‐skin contact compared to no treatment or control likely leads to a large reduction in PIPP at 30 s after heel prick (MD −3.47 [−5.55, −1.38], moderate‐certainty, Appendix S6). The Cochrane review also included an RCT that reported PIPP at 30 s favoring the skin‐to‐skin group compared to control, but data were presented on an ordinal scale so could not be included in the meta‐analysis.

The Johnston et al. [29] Cochrane review suggested that skin‐to‐skin contact compared to no treatment may result in little to no difference in PIPP score at 60 s (MD −0.33 [−2.62, 1.97], low‐certainty) and 90 s (MD −0.77 [−1.69, 3.22], moderate‐certainty) after heel prick but probably increased PIPP score at 120 s after heel prick (MD 1.41 [−0.37, 3.19], moderate‐certainty). Additionally, an RCT reported reduced PIPP at 1 and 2 min after the painful procedure, but it could not be added to the meta‐analysis as no standard deviations were reported.

In the Johnston et al. [29] Cochrane review, one RCT reported reduced pain during recovery in the skin‐to‐skin group compared to control, but raw data were not provided. Two RCTs not included in the Cochrane review, Ding et al. [49] and Fu [51] were meta‐analyzed and showed that skin‐to‐skin compared to control likely reduced NFCS 30 s after heel prick (MD −0.78 [−0.95, −0.60], moderate‐certainty) (Appendix S6).

Two additional RCTs Liao 2020 [52] and Shi 2017 [53] using NIPS scores after heel prick were meta‐analyzed and showed that skin‐to‐skin contact reduces NIPS score after heel prick compared to control (MD −1.24 [−1.48, −1.00], moderate‐certainty).

*Unclear whether during or after procedure:* In the Johnston et al. [29] Cochrane review, one RCT reported that DAN values favored skin‐to‐skin contact compared to control but did not report raw data. A more recent RCT not included in the Cochrane review also reported that skin‐to‐skin contact likely reduced DAN values compared to control (MD −1.54 [−1.85, −1.23], moderate‐certainty) [54].

##### 3.1.2.2 Secondary Outcomes

###### 3.1.2.2.1 Heart Rate

The evidence in the Johnston et al. [29] Cochrane review is very uncertain about the effect of skin‐to‐skin contact on heart rate during the painful procedure compared to control or additional swaddling (MD –10.78 bpm [−13.63, −7.93]). Several RCTs in the review also reported heart rate data during the painful procedure that could not be included in the meta‐analysis. Two trials found no difference between the skin‐to‐skin and control groups, whilst one found lower maximum heart rate and one lower median heart rates in the skin‐to‐skin group.

According to the Johnston et al. [29] Cochrane review, skin‐to‐skin contact compared to control resulted in little to no difference in heart rate following the painful procedure (MD 0.08 bpm [−4.39, 4.55], high‐certainty). Again, several RCTs did not provide sufficient data to be included in the meta‐analysis, so they were reported narratively. One reported lower heart rate in the skin‐to‐skin group compared to control, and one reported reduced time to return to baseline heart rate after blood sampling.

###### 3.1.2.2.2 Oxygen Saturation

According to the Johnston et al. [29] Cochrane review, skin‐to‐skin contact compared to control may increase oxygen saturation during the painful procedure at 30 s (MD 1.73% [−0.53, 3.99], low‐certainty), but evidence is very uncertain at 60 s (MD 2.17% [−0.12, 4.46]). Of the additional RCTs, one reported no significant difference in oxygen saturation and two found no statistically significant difference in the change in oxygen saturation between groups after heel prick, but none provide sufficient data for meta‐analysis.

###### 3.1.2.2.3 Cortisol

Compared with the control group, one study in the Johnston et al. [29] Cochrane review found higher salivary cortisol concentrations in the group that received 80 min of skin‐to‐skin contact but lower concentrations in the group that received 30 min.

###### 3.1.2.2.4 Duration of Crying

According to the Johnston et al. [29] Cochrane review, skin‐to‐skin contact compared to control likely reduced the duration of crying during heel prick (MD −34.16 s [−42.86, −25.45], moderate‐certainty). Two other heel prick RCTs did not provide sufficient data for meta‐analysis but found significantly reduced levels of crying with skin‐to‐skin contact.

### 3.2 Promising Interventions

#### 3.2.1 Breastfeeding [33]

##### 3.2.1.1 Primary Outcome

Compared to no intervention, breastfeeding likely results in a large reduction in NIPS score (MD –2.53 [−3.46, −1.60], moderate‐certainty). It also may result in a large reduction in NFCS score (MD –4.20 [−5.14, −3.26], low‐certainty) and a reduction in DAN score (MD –1.87 [−4.61, 0.86], low‐certainty). However, breastfeeding may result in little to no difference in PIPP score compared to no intervention (MD –0.49 [−2.39, 1.41], low‐certainty). Compared to placebo, it may lead to a large reduction in PIPP score (MD –5.95 [−7.42, −4.48], low‐certainty) and a large reduction in DAN score (MD –6.24 [−7.38, −5.10], low‐certainty).

##### 3.2.1.2 Secondary Outcomes

Compared to control, breastfeeding may result in a reduction in heart rate (MD –5.56 bpm [−16.34, 5.22], low‐certainty). Breastfeeding may result in a reduction in heart rate change during the painful procedure (MD –12.89 bpm [−19.93, −5.85], low‐certainty) compared to no intervention, but little to no difference in oxygen saturation (MD 0.64% [0.21, 1.08], low‐certainty) or oxygen saturation change (MD –0.04% [−1.32, 1.24], low‐certainty).

Two RCTs lacking standard deviations were not suitable for meta‐analysis because standard deviations were not provided; this suggested breastfeeding may reduce heart rate changes with little or no effect on oxygen saturation.

Breastfeeding may reduce systolic blood pressure change compared to no intervention (MD –5.48 mmHg [−12.28, 1.32], low‐certainty), but there was little to no difference in diastolic blood pressure change (MD –0.92 mmHg [−5.58, 3.74], low‐certainty).

Breastfeeding may increase the latency to first cry (MD 8.82 s [7.98, −9.66], low certainty) and reduce the duration of first cry (MD –18.00 s [−25.80, −10.20], low‐certainty) and likely reduce the duration of crying (MD –36.23 s [−55.57, −16.89], moderate‐certainty).

Carbajal et al. [55] reported that there was no difference in the number of neonates with effective sucking between the breastfeeding and placebo groups 48–72 h after venipuncture.

#### 3.2.2 Supplemental Breast Milk [33]

##### 3.2.2.1 Primary Outcome

Compared to one or two doses of water, supplemental breast milk may reduce NFCS score at 2 min (MD –0.84 [−1.09, −0.59], MD –0.59 [−0.83, −0.35] respectively, both low‐certainty). Compared to water, supplemental breast milk may reduce NFCS score at 3 min (MD –0.86 [−2.24, 0.52], low‐certainty) but had little to no effect on body pain score (MD 0.48 [−0.38, 1.34], low‐certainty). “Body pain score” appears to refer to the Infant Body Coding System, which scores movement as present or absent in the hands, feet, arms, legs, head and torso [56].

Compared to no intervention, supplemental breast milk may reduce DAN score (MD −1.00 [−2.15, 0.15], low‐certainty) but had little to no effect on NIPS score (MD −0.30 [−1.60, 1.00], low‐certainty) and N‐PASS score (−0.45 [−0.55, −0.35], low‐certainty).

##### 3.2.2.2 Secondary Outcomes

Compared to no intervention, supplemental breast milk may reduce heart rate (MD −20.00 bpm [−28.74, −11.26], low‐certainty), and respiratory rate (MD −10.90 rpm [−14.70, −7.10], low‐certainty), and increase oxygen saturation (MD 6.00% [4.07, 7.93], low‐certainty). No RCTs reported on these outcomes for supplemental breast milk compared to placebo.

Compared to no intervention, supplemental breast milk may result in less change in heart rate (MD –4.81 bpm [−6.28, −3.35], low‐certainty) and less change in oxygen saturation change (MD –4.90% [−5.98, −3.82], low‐certainty). Compared to placebo, supplemental breast milk likely results in reduced heart rate change (MD –3.13 bpm [−9.51, 3.26], moderate‐certainty) and may result in little to no difference in oxygen saturation change (MD –0.92% [−2.47, 0.63], low‐certainty). Two RCTs reported heart rate change but lacked data for meta‐analysis; one found reduced heart rate change with colostrum on a pacifier versus placebo, and the other found no difference between supplemental breast milk and no intervention. Another RCT found no difference in oxygen saturation change between supplemental breast milk and control but provided no data.

Compared to placebo, supplemental breast milk may result in little to no effect in systolic blood pressure change (MD 1.10 mmHg [−2.93, 5.13], low‐certainty) and diastolic blood pressure change (MD −0.50 mmHg [−5.18, 4.18], low‐certainty).

Supplemental breastmilk may increase the duration of crying compared to no intervention (MD 36.70 s [0.60, 72.80], low‐certainty) but likely reduced the duration of crying compared to placebo (MD –8.67 s [−12.32, −5.02], moderate‐certainty). It may increase the percentage of time crying compared to placebo (MD 9.00 [−1.99, 19.99], low‐certainty). An additional RCT reported narratively found no difference in the percentage of time crying between the group given colostrum (via pacifier or syringe) and the control group.

One study in the Shah et al. [33] systematic review reported no difference in desaturation, nausea, regurgitation, vomiting, bradycardia, or tachycardia between supplemental breast milk and placebo.

#### 3.2.3 Acupuncture [27]

##### 3.2.3.1 Primary Outcome

Acupuncture may reduce pain compared to no intervention during the painful procedure (SMD −0.56, [−0.75, −0.37], low‐certainty), up to 10 min after the procedure (SMD –0.58 [−0.82, −0.34], low‐certainty) and 1–2 h after the procedure (MD –1.29 [−2.07, −0.51], low‐certainty).

##### 3.2.3.2 Secondary Outcomes

Acupuncture may result in little to no difference in any harms compared to no intervention (RR 0.35, [0.01, 8.31], low‐certainty).

#### 3.2.4 Light Reduction [32]

These interventions aimed to reduce environmental light exposure through direct or indirect light shielding to minimize stress and pain responses.

##### 3.2.4.1 Primary Outcome

In preterm neonates, light reduction likely reduced pain reactivity (SMD –0.71 [−1.08, −0.34], moderate‐certainty) and improved immediate pain regulation compared to a no‐treatment control (SMD –1.16 [−1.53, −0.78], moderate‐certainty).

#### 3.2.5 Breast Milk Odor [38]

##### 3.2.5.1 Primary Outcome

In preterm neonates, breast milk odor may reduce pain scores compared with no odor stimulation (SMD −0.95 [−1.45, −0.45]; low‐certainty). The effect was observed in both venipuncture (MD −3.19 [−4.15, −2.24]) and heel prick procedures (MD −0.58 [−0.89, −0.27]).

##### 3.2.5.2 Secondary Outcomes

In preterm neonates, breast milk odor may increase oxygen saturation (MD 2.4 [0.68, 4.12]; low‐certainty) and may reduce heart rate (MD −9.26 [−14.28, −4.24]; moderate‐certainty).

### 3.3 Interventions With Inconclusive Evidence

#### 3.3.1 Opioids [30]

Of the seven included RCTs that compared opioid use (primarily morphine, fentanyl and remifentanil) to control, only one was a blood sampling study and another included both heel prick and retinopathy of prematurity screening.

##### 3.3.1.1 Primary Outcome

*During the Procedure:* Compared to placebo, opioids likely result in a large reduction in PIPP/PIPP‐R scores during the painful procedure (MD –2.58 [−3.12, −2.03], moderate‐certainty).

*After the Procedure:* The evidence is very uncertain about the effect of opioids on PIPP/PIPP‐R 1–2 h after the procedure (MD −0.83, [−2.42, 0.75]). Opioids may result in little to no effect in DAN score 1–2 h after the procedure (MD –0.20 [−2.21, 1.81], low‐certainty).

##### 3.3.1.2 Secondary Outcomes

Compared to placebo, opioids may increase episodes of apnoea (RR 3.15 [1.08, 9.16], low‐certainty). The evidence is very uncertain about the effect of opioids on the rate of severe apnoea requiring resuscitation (RR 7.44 [0.42, 132.95]), episodes of bradycardia (RR 3.19 [0.14, 72.69]), episodes of desaturation (RR 1.82 [0.72, 4.58]), and hypotension (RR not estimable due to no events).

#### 3.3.2 Paracetamol [31]

##### 3.3.2.1 Primary Outcome

Compared to placebo, paracetamol may increase the difference in PIPP score between baseline and the heel prick period (MD 1.40 [−0.45, 3.25], low‐certainty). Paracetamol may result in little to no difference in maximum PIPP or NIPS within 3 min of heel prick (MD 1.48 [−0.11, 3.07], MD 0.85 [−0.14, 1.84] respectively, both low‐certainty).

##### 3.3.2.2 Secondary Outcomes

Compared to placebo, paracetamol may increase the difference in oxygen saturation between baseline and the heel prick period (MD 2.60% [−0.58, 5.78], low‐certainty). It may have little to no effect on the difference in heart rate between baseline and heel prick (MD 1.90 bpm [−4.74, 8.54], low‐certainty).

Paracetamol may result in little to no difference in duration of crying compared to placebo (MD 8 s [−19, 35], low‐certainty).

#### 3.3.3 Opical Anesthetics [28]

##### 3.3.3.1 EMLA

*Primary Outcome:* Several RCTs reported the effect of a eutectic mixture of local anesthetics (EMLA) on pain scores, but due to differences in outcomes these could not be combined. Compared to placebo, EMLA may result in little to no effect in PIPP (MD 0.27 [−1.45, 1.99], low‐certainty) or NIPS score (MD 0.27 [−0.75, 1.29], low‐certainty).

One RCT also used PIPP score and reported no statistically significant difference between EMLA applied for 30 or 60 min and placebo during heel lancing, but the data could not be meta‐analyzed due to use of a modified scale for neonates < 28 weeks' gestation. Another RCT reported lower NFCS scores in the EMLA group than the placebo group at 0–15 s after venepuncture, but no difference by 60–70 s.

*Secondary Outcomes:* One RCT reported no significant difference in total cry duration between EMLA and placebo groups but did not report raw data. EMLA may result in a large increase in short‐lasting skin reactions compared to placebo (RR 1.65 [1.24, 2.19], low‐certainty).

##### 3.3.3.2 Amethocaine

*Primary Outcome:* Two RCTs assessed neonates during venipuncture and reported a greater proportion of neonates with little or no pain response (NFCS ≤ 10 in 5 s) with amethocaine compared to placebo.

*Secondary Outcomes:* One RCT reported no significant difference in cry duration between amethocaine and placebo groups. Two RCTs reported no local skin reactions in either group.

#### 3.3.4 Music‐Based Interventions [39]

*Primary Outcome:* Results of the Costa et al. [39] systematic review were presented narratively. Although the review included both music medicine and music therapy interventions, only music medicine interventions met the inclusion criteria for this overview because music therapy studies did not involve blood sampling procedures. Three RCTs that assessed music medicine interventions during blood sampling reported reduced pain scores in the music groups compared to control. Another RCT reported reduced pain scores with recorded music that mothers listened to during the last trimester of pregnancy compared to control, but no difference in listening to other recorded music. One RCT reported reduced pain scores compared to control only with recorded music and kangaroo care, not with recorded music alone. Two other RCTs reported reduced pain scores in the recorded music group compared to control, but the music groups received co‐interventions including touch or non‐nutritive sucking, facilitated tucking, and holding.

*Secondary Outcomes:* In the Costa et al. [39] systematic review, one RCT reported reduced heart rate increase and increased oxygen saturation in the recorded music group compared to control. Another RCT reported reduced heart rate in the recorded music group compared to control. Other RCTs reported no difference between groups for oxygen saturation (3 RCTs), heart rate (2 RCTs), respiratory rate (2 RCTs), and blood cortisol (1 RCT).

In this systematic review, two RCTs had behavioral outcomes reported. One showed an improved sleep–wake state in the recorded music group compared to control, with these infants more likely to be sleeping or relaxed. The other reported increased time in a quiet state with recorded music that mothers listened to during the last trimester of pregnancy compared to control, but no difference listening to other recorded music.

#### 3.3.5 Other Non‐Pharmacological Interventions

The rest of the interventions with evidence are from the Cochrane review of non‐pharmacological interventions [32]. This review combined different pain scores and calculated a standardized mean difference. They defined pain reactivity as pain scores in the first 30 s post‐procedure and immediate pain regulation as pain scores from 30 s–5 min post‐procedure. They also separated outcomes in preterm and term neonates.

##### 3.3.5.1 Non‐Nutritive Sucking [32]

*Primary Outcome:* In preterm neonates, the evidence is very uncertain about the effect of non‐nutritive sucking compared to no‐treatment control on pain reactivity (SMD −0.57 [−1.03, −0.11]) and immediate pain regulation (SMD −0.61 [−0.95, −0.27]).

Similarly, in term neonates, the evidence is very uncertain about the effect of non‐nutritive sucking on pain reactivity (SMD –1.13 [−1.57, −0.68]) and immediate pain regulation (SMD –1.49 [−2.20, −0.78]) compared to no‐treatment control.

*Secondary Outcomes:* Evidence is also very uncertain about the effect of non‐nutritive sucking on adverse events, including vomiting in preterm neonates and desaturation in term neonates. No adverse events in either group were reported by 15 studies.

##### 3.3.5.2 Swaddling [32]

*Primary Outcome:* In preterm neonates, the evidence is very uncertain about the effect of swaddling compared to no‐treatment control on pain reactivity (SMD –0.60 [−1.23, 0.04]) and immediate pain regulation (SMD –1.21 [−2.05, −0.38]).

In term neonates, swaddling may lead to a reduction in pain reactivity (SMD –0.89 [−1.24, −0.55], with low‐certainty evidence) and may improve immediate pain regulation (no raw data reported, low‐certainty).

*Secondary Outcomes:* In preterm and term neonates, no adverse events were reported in either group. Thus, swaddling may result in little to no difference in adverse events, with low‐certainty and very‐low‐certainty evidence respectively.

##### 3.3.5.3 Facilitated Tucking [32]

*Primary Outcome:* In preterm neonates, the evidence is very uncertain about the effect of facilitated tucking compared to no‐treatment control on pain reactivity (SMD –1.01 [−1.44, −0.58]) and immediate pain regulation (SMD –0.59 [−0.92, −0.26]).

*Secondary Outcome:* In preterm neonates, the evidence is very uncertain about the effect of facilitated tucking on adverse events compared to a no‐treatment control. Of 10 included studies, one reported one incidence of septicaemia after receiving facilitated tucking and others reported no adverse events.

##### 3.3.5.4 Swallowing Water [32]

*Primary Outcome:* In preterm neonates, swallowing water compared to no‐treatment control may result in little to no difference in pain reactivity (SMD 1.10 [−1.56, 3.75], low‐certainty evidence) and immediate pain regulation (no raw data reported, moderate‐certainty).

##### 3.3.5.5 Rocking or Holding [32]

*Primary Outcome:* In term neonates, the evidence is very uncertain about the effect of rocking or holding on pain reactivity (SMD –0.09 [−0.61, 0.43]) and immediate pain regulation (SMD –0.84 [−1.15, −0.53]) compared to a no‐treatment control.

*Secondary Outcome:* In term neonates, no adverse events were reported in either the rocking/holding or no‐treatment control group (no raw data provided, moderate‐certainty).

##### 3.3.5.6 Touch/Massage‐Related [32]

These interventions included tactile stimulation approaches such as massage, therapeutic touch, pressure stimulation, or facilitated tactile contact designed to provide counter‐stimulation to painful input. However, the original systematic review did not report who delivered these interventions.

*Primary Outcome:* In preterm neonates, the evidence is very uncertain about the effect of touch/massage‐related interventions compared to a no‐treatment control on pain reactivity (SMD –0.92 [−1.60, −0.25]), but these interventions may improve immediate pain regulation (SMD –1.24 [−1.85, −0.63], low‐certainty).

In term neonates, touch/massage‐related interventions compared to a no‐treatment control may improve immediate pain regulation (SMD –0.81 [−1.22, −0.40], low‐certainty), but the evidence is very uncertain about the effect of touch/massage‐related interventions (SMD –0.92 [−1.57, −0.28]) on immediate pain regulation.

*Secondary Outcome:* In preterm and term neonates, there were no adverse events reported in either touch/massage‐related or no‐treatment control groups (moderate‐certainty and low‐certainty respectively).

##### 3.3.5.7 Sound Reduction [32]

These interventions aimed to reduce environmental noise exposure to minimize stress and pain reactivity in hospitalized infants.

*Primary Outcome:* In preterm neonates, the evidence is very uncertain about the effect of sound reduction compared to a no‐treatment control on pain reactivity (SMD –0.64 [−2.06, 0.78]) and immediate pain regulation (no raw data reported).

##### 3.3.5.8 Sound Addition [32]

These interventions involved exposure to soothing auditory stimuli designed to simulate the fetal environment, including recorded maternal speaking voice, recorded intrauterine sounds (e.g., heartbeat), or other recorded soothing environmental sounds.

*Primary Outcome:* In preterm neonates, the evidence is very uncertain about the effect of sound addition compared to a no‐treatment control on pain reactivity (SMD –0.89 [−1.43, −0.34]), but it may improve immediate pain regulation (SMD –0.92 [2.20, 0.36], low‐certainty).

In full term neonates, the evidence is very uncertain about the effect of sound addition on pain reactivity compared to a no‐treatment control (insufficient data reported for meta‐analysis). No data were reported assessing immediate pain regulation in full term neonates.

*Secondary Outcomes:* In preterm neonates, the evidence is very uncertain about the effect of sound addition on adverse events compared to a no‐treatment control (no events). No data were reported assessing adverse events in full term neonates.

##### 3.3.5.9 Smell Addition [32]

These interventions involved exposure to soothing smells during procedures, including maternal odor, breastmilk odor, lavender, or vanilla. Some breastmilk odor trials overlapped with those included in the separate breastmilk odor section above. We retained these studies in both sections because the original review combined odor‐based interventions, preventing the isolation of breastmilk odor trials from the pooled analysis.

*Primary Outcome:* In preterm neonates, the evidence is very uncertain about the effect of smell addition compared to a no‐treatment control on pain reactivity (SMD –0.41 [−0.72, −0.10]) and immediate pain regulation (SMD –0.57 [−0.88, −0.27]).

In term neonates, the evidence is very uncertain about the effect of smell addition on pain reactivity (SMD –0.77 [−1.09, −0.44]) compared to a no‐treatment control. Smell addition likely slightly improves immediate pain regulation (SMD –0.41 [−0.80, −0.01], moderate‐certainty). One study found that familiar smells improved immediate pain regulation in comparison to no treatment, but unfamiliar smells did not.

*Secondary Outcome:* In preterm and term neonates, no adverse events were reported in either smell addition or no‐treatment control groups (moderate‐certainty and low‐certainty respectively).

##### 3.3.5.10 Heat Addition [32]

These interventions involved warming the procedural site, such as heel warming before heel lance, using non‐pharmacological warming methods.

*Primary Outcome:* In preterm neonates, the evidence is very uncertain about the effect of heat addition on pain reactivity (no raw data reported). No data were reported on immediate pain regulation in preterm neonates.

In term neonates, heat addition may result in little to no difference in pain reactivity compared to a no‐treatment control (SMD –0.12 [−0.42, 0.17], moderate‐certainty). No data were reported on immediate pain regulation in term neonates.

*Secondary Outcome:* In both preterm and term neonates, no adverse events were reported in heat addition or no‐treatment control groups (very‐low certainty).

##### 3.3.5.11 Cold Addition [32]

These interventions involved cooling the procedural site using non‐pharmacological methods such as cold packs.

*Primary Outcome:* In term neonates, cold addition may reduce pain reactivity compared to a no‐treatment control (SMD –0.85 [−1.48, −0.23], low‐certainty).

##### 3.3.5.12 Multisensory Bundle [32]

These interventions combined multiple analgesic strategies targeting different sensory pathways, including combinations of touch, smell, sound, swaddling, non‐nutritive sucking, or environmental modifications.

*Primary Outcome:* In preterm neonates, the evidence is very uncertain about the effect of a multisensory bundle on pain reactivity (SMD –2.70 [−4.16, −1.23]) and immediate pain regulation (SMD –1.49 [−2.92, −0.06]) compared to a no‐treatment control. In term neonates, a multisensory bundle likely results in little to no difference in pain reactivity compared to a no‐treatment control (SMD 0.00 [−0.29, 0.30], moderate‐certainty). No data reported immediate pain regulation in term neonates.

*Secondary Outcome:* In both preterm and term neonates, no adverse effects were detected for either a multisensory bundle or no‐treatment control groups (low‐certainty and very‐low‐certainty respectively).

##### 3.3.5.13 Therapeutic Touch [32]

This involves holding one's hands over the neonate without direct contact, based on the integrative field of energy medicine. No studies compared therapeutic touch to a no‐treatment control in term neonates.

*Primary Outcome:* In preterm neonates, evidence was very uncertain about the effect of therapeutic touch on pain reactivity and pain regulation (no raw data reported).

##### 3.3.5.14 Co‐Bedding for Twins [32]

*Primary Outcome:* In preterm neonates, co‐bedding may not reduce pain reactivity compared to a no‐treatment control (no raw data, low‐certainty).

*Secondary Outcome:* In preterm neonates, no adverse events were reported in the co‐bedding or no‐treatment control groups (no raw data, low‐certainty).

### 3.4 Interventions With no Eligible Studies

#### 3.4.1 Non‐Opioid Analgesics

The Cochrane systematic review on non‐opioid analgesics did not include any studies that involved blood sampling procedures [36].

#### 3.4.2 Clonidine

The Cochrane systematic review on clonidine for pain in non‐ventilated neonates found no studies eligible for inclusion [37].

### 3.5 Network Meta‐Analysis (NMA) Evidence (Comparative Effectiveness)

Abiramalatha [40], including 103 RCTs, found high‐certainty evidence that non‐nutritive sucking combined with sucrose was the most effective intervention, followed by breastfeeding, glucose, expressed breastmilk, and sucrose. Combination interventions also showed benefit but with variable certainty.

Amuji [41], including 37 RCTs, found that parent‐led interventions were effective for venipuncture pain. Breastfeeding (including expressed breastmilk) ranked highest, followed by non‐nutritive sucking, both supported by high‐certainty evidence. However, glucose and swaddling showed only low‐certainty benefit, with substantial overlap with pairwise reviews.

Ding [42], including 28 RCTs, found that multiple acoustic interventions (e.g., recorded maternal voice, music‐based interventions, white noise) reduced pain during and immediately after procedures. White noise combinations ranked highest during procedures, while music‐based combinations ranked highest post‐procedure. However, the effects were not sustained beyond 1 min, and the certainty of the evidence was not assessed.

Efendi [43], including 42 RCTs, showed that glucose and sucrose reduced procedural pain across phases, with expressed breastmilk also showing benefit. Glucose ranked highest but was associated with increased adverse events, highlighting a benefit–harm trade‐off.

Hasanul Huda [44], including 22 RCTs, reported that skin‐to‐skin contact was the only intervention with a statistically significant reduction in pain compared with usual care (moderate certainty). Other interventions, including white noise and massage, ranked highly but were not statistically superior. Overall certainty remained limited.

Shen [45] and Tao [46] evaluated multisensory interventions using largely overlapping evidence bases. Both identified tactile‐dominant combinations as most effective, with variation by procedure type. Shen 2025 reported tactile–kinesthetic stimulation as most effective overall, while Tao found tactile–taste–kinesthetic and tactile–taste–auditory combinations most effective for different sampling methods. Examples of these combinations included facilitated tucking or touch combined with sucrose/glucose and additional auditory stimulation such as recorded maternal voice, recorded music, or white noise. Despite methodological differences, both analyses consistently supported tactile‐based multisensory interventions as the most effective subgroup. However, neither review formally assessed certainty of evidence.

## 4 Discussion

### 4.1 Summary of Main Results

This overview synthesized 22 systematic reviews, including pairwise meta‐analyses and network meta‐analyses on pain management during blood sampling. High‐certainty evidence supports sucrose, and moderate‐certainty evidence supports skin‐to‐skin contact for neonatal pain management. Breastfeeding, expressed breastmilk, supplemental breast milk, glucose, acupuncture and breast milk odor also appeared beneficial, although certainty of evidence was generally low to moderate and varied across outcomes and intervention combinations. NMAs consistently suggested that tactile parent‐led interventions (e.g., skin‐to‐skin contact and breastfeeding), oral sweet solutions (sucrose/glucose), and selected multisensory combinations were among the most effective approaches for neonatal procedural pain management. However, interpretation of indirect comparative findings should be cautious because of substantial overlap in primary studies, heterogeneity in intervention classification and outcome measurement, methodological limitations in included NMAs, and generally low to moderate certainty of evidence for many interventions.

A previous overview by Qiao et al., 2022 examined interventions for procedural pain in neonates, with a search period from November 2016 to November 2021. As a result, it did not include the recent updates of several Cochrane reviews, including sucrose for heel‐prick [35], sucrose for venipuncture [26], breastfeeding or breastmilk [33] and non‐pharmacological management [32]. Despite several methodological differences, Qiao 2022 also suggested that sucrose and skin‐to‐skin reduced pain scores during painful procedures. Unlike our overview, the Qiao et al., 2022 overview [57], did not include opioids [30], paracetamol [31] or topical anesthetics [28].

### 4.2 Possible Mechanisms

Evidence from animal studies suggests that sucrose analgesia is primarily mediated by sweet taste induced release of β‐endorphins, which activate opioid pathways and reduce pain [58, 59]. Other potential pathways involved include stimulation of dopamine and acetylcholine release.

The analgesic effect of skin‐to‐skin contact likely results from sensory saturation, stimulation of c‐afferent nerves, oxytocin release [60] and parent–infant co‐regulation. Oxytocin increases parasympathetic nervous system activity and β‐endorphin concentrations, contributing to pain relief [61]. These effects are particularly important in neonates, whose descending inhibitory pain pathways are immature and rely on external sensory input for modulation [62].

Mechanisms of the analgesic effect of breastfeeding are thought to include skin‐to‐skin contact between mother and baby, transfer of heat, rocking, co‐regulation, slightly sweet taste and the mother's sound and smell [63]. In contrast, the analgesic effects of breastmilk odor are likely mediated through familiar olfactory cues associated with feeding and maternal presence, which may reduce stress and promote infant calming. Additionally, breastmilk contains natural endorphins and high concentrations of tryptophan. Tryptophan is converted to melatonin which may increase concentrations of β‐endorphins. The slight sweetness of breastmilk is thought to contribute little to pain relief, as breastmilk only contains approximately 7% lactose, the least sweet sugar. The analgesic effect of supplemental breastmilk may therefore involve both sensory and biological mechanisms, including the endorphins present in breastmilk. Light reduction may have an analgesic effect through reducing stress in neonates. This may make the brain less sensitive to the effects of painful interventions [32].

These mechanisms likely act in complementary ways, particularly for multi‐sensory interventions such as skin‐to‐skin contact, breastfeeding, and breastmilk odur. Neonatal pain is increasingly recognized as a complex biopsychosocial experience, and effective interventions may work through overlapping biological, sensory, emotional, and relational pathways. The biological plausibility and consistency of these mechanisms across studies strengthen confidence in their observed effectiveness, supporting the moderate to high certainty for interventions like sucrose and skin‐to‐skin contact.

### 4.3 Strengths and Limitations of This Overview

#### 4.3.1 Overall Completeness and Applicability of Evidence

The evidence included in this overview directly addresses some of the pre‐specified outcomes, including validated pain scores and adverse effects. Strengths include the amount of evidence largely included in up‐to‐date Cochrane reviews, together with the addition of recent NMAs that expand the comparative evidence base across multiple interventions. However, no reviews reported cerebral oxygenation as measured by near‐infrared spectroscopy, parent satisfaction, clinician satisfaction or cost of the intervention. Parent and clinician satisfaction are important for the successful implementation of pain management strategies. Equity is also an important consideration in neonatal pain management, and many of the effective or promising interventions, such as skin‐to‐skin contact, breastfeeding and light reduction are low‐cost and feasible in low‐resource settings.

Recent NMAs broadened the comparative evidence base by synthesizing multiple intervention classes simultaneously and generally supported findings from pairwise reviews. However, comparative rankings should be interpreted cautiously because of substantial overlap in primary studies, heterogeneity in intervention classification and outcome assessment, inconsistent certainty assessments, and methodological limitations across several NMAs, including inappropriate study‐level application of ROB 2. Given the well‐established evidence that neonates experience pain and that several interventions are effective for reducing procedural pain, the continued use of placebo, water, or no‐treatment control groups in neonatal pain trials warrants careful ethical consideration. Future research may be better directed toward comparative effectiveness studies between active interventions, evaluation of multimodal approaches, and implementation strategies to improve uptake of evidence‐based pain management in clinical practice.

Several of the included systematic reviews highlighted the limited evidence in very preterm infants. Foster et al. [28] found no evidence in very preterm neonates, whilst Shah et al. [33] reported that most included studies included healthy term neonates or stable late preterm neonates. They highlighted the need for a suitable pain management strategy for very preterm or sick neonates, who experience repeated painful procedures, including blood sampling. Although some of the reviews reported evidence stratified for preterm and term infants where possible [32, 34, 35], most did not, meaning conclusions could not be drawn about efficacy across different gestational ages.

Evidence for breastfeeding, opioids, paracetamol, topical anesthetics, non‐nutritive sucking, swaddling, facilitated tucking, swallowing water, touch/massaged related, sound addition, smell addition, light reduction, cold addition, and multisensory bundle often included painful procedures other than blood sampling, primarily intramuscular injection, but also included more painful interventions, such as lumbar puncture, retinopathy of prematurity screening or endotracheal suctioning. To maintain focus, only blood sampling studies were included in the skin‐to‐skin Cochrane review updates and narratively, aligning with the scope of this overview. Music‐based interventions included in this overview were limited to music medicine approaches during blood sampling procedures, primarily involving recorded music and sound exposure interventions, and therefore may not be generalisable to broader music therapy interventions used in neonatal care.

There was considerable variability across studies in the choice of outcome measures, pain scoring systems, and timing of assessments. This heterogeneity complicates direct comparisons and meta‐analyses, potentially limiting the precision and generalisability of conclusions drawn from the evidence.

#### 4.3.2 Quality of the Evidence

The methodological quality of the included pairwise systematic reviews was generally moderate to high. Based on AMSTAR 2 assessments, eight reviews were rated as high quality, three as moderate quality, and one as critically low quality. All reviews included RCTs and/or quasi‐RCTs, clearly defined their research questions and inclusion criteria using PICO components, and used appropriate methods for statistical synthesis where meta‐analysis was conducted. Most reviews also performed study selection in duplicate, adequately described included studies, assessed risk of bias of included studies using appropriate methods, investigated publication bias where applicable, and reported potential conflicts of interest and funding sources of the review authors.

The most common methodological limitations were failure to report funding sources of included primary studies, lack of duplicate data extraction, absence of a list of excluded studies with justification, inadequate consideration of the impact of risk of bias in primary studies on meta‐analysis results, insufficient incorporation of risk of bias when interpreting findings, and limited explanation of heterogeneity. One review was rated critically low quality because of multiple critical methodological weaknesses, including absence of a pre‐established protocol, inadequate literature searching, lack of duplicate data extraction, limited reporting of excluded studies, and inadequate consideration of risk of bias and heterogeneity in the interpretation of findings.

Most NMAs conducted comprehensive literature searches, assessed inconsistency and heterogeneity, and used structured approaches such as GRADE for NMA or CINeMA to evaluate certainty of evidence. However, six of the seven NMAs applied ROB 2 at the study level by assigning overall study judgments rather than conducting outcome‐specific assessments as recommended by ROB 2 guidance. In addition, three NMAs did not formally assess certainty of evidence. Common methodological concerns across NMAs included substantial overlap in primary studies, heterogeneity in intervention classification, and generally low to moderate certainty of evidence for many intervention comparisons.

#### 4.3.3 Quality of the Overview

This overview is comprehensive, including both pharmacological and non‐pharmacological interventions. This allows a wide range of intervention options to be considered for families, depending on the circumstance. We updated the 2017 skin‐to‐skin Cochrane review, as many new studies had been released since its publication. This meant that the conclusions in this review are based on up‐to‐date evidence. We also assessed a wide range of secondary outcomes, including physiological and behavioral outcomes that can indicate pain. This allows us to gain a more complete picture of neonatal pain, as interventions may improve physiological outcomes and not behavioral outcomes, or vice versa [64].

However, the certainty of evidence was very low for many interventions and outcomes, particularly non‐pharmacological interventions. As mentioned by Pillai‐Riddel et al. [32], blinding is difficult in these studies, with the lack of blinding of personnel and outcome assessors the reason that 69 of the 103 studies at high risk of bias in this review (total 138 studies) were rated high risk. They suggested that tools assessing risk of bias in non‐pharmacological studies should differentiate between study personnel and outcome assessors being blinded to the study hypothesis, which they deemed important, and being blinded to the intervention, which was largely unfeasible.

There have been many reviews conducted on pain management strategies in neonates, some focussing on specific strategies and others having broader inclusion criteria. Many of these reviews included some studies that overlapped with other systematic reviews and others that did not. Because overlapping reviews had to be excluded to avoid double counting of data, this meant we could not ensure that all relevant studies contributed evidence to this overview. The alternative of extracting all studies from every review and combining them manually was not feasible. Therefore, despite this limitation, our approach made it possible to synthesize the large quantity of information on this important topic.

The results of this overview included pain measured on many different scales, making results difficult to interpret. This is because many of the reviews included chose not to combine scores from different pain scales into a single outcome. Even when the same pain scale was used, often they were used so differently that review authors decided not to combine the results [29]. Some study authors did not provide sufficient raw data for inclusion in meta‐analysis. This highlights the need for standardized reporting sets for future research, so that findings can be synthesized to provide more robust evidence. Review authors also may have differed in how they assessed GRADE certainty of evidence and the magnitude of effect of interventions.

### 4.4 Clinical Implications

This overview highlights a range of evidence‐informed options for procedural pain management in neonates. Given the variable certainty of evidence, clinicians should consider both effectiveness and feasibility when selecting strategies, including contextual factors such as gestational age, type of procedure, family preferences, and resource availability. Neonatal procedural pain management should be considered within a family‐centred and family‐integrated care framework that supports parental involvement in decision‐making, pain assessment, and delivery of parent‐led interventions such as skin‐to‐skin contact, breastfeeding, touch, and voice. Successful implementation requires supportive organizational structures, clinician engagement, and policies that empower and prepare parents to participate in neonatal pain care. These approaches may be particularly suited to low‐resource or bedside care settings.

## 5 Conclusion

This overview supports the use of several effective strategies for neonatal procedural pain management during blood sampling, particularly parent‐led and other non‐pharmacological interventions such as skin‐to‐skin contact and breastfeeding. Given their low risk, accessibility, and broader biopsychosocial benefits, non‐pharmacological parent‐led interventions should be considered first‐line approaches for neonatal procedural pain management where feasible. Future research priorities should shift from repeatedly evaluating intervention efficacy toward implementation, optimization, and equitable access to evidence‐based family‐centered pain management strategies.

## Funding

This work was supported by the Health Research Council of New Zealand (19/690) and Aotearoa Foundation (9909494).

## Conflicts of Interest

The authors declare no conflicts of interest.

## Supporting information


**Appendix S1:** Protocol for overeview of pain management strategies, registered prospectively on PROSPERO (CRD42023474078).
**Appendix S2:** Search Strategies.
**Appendix S3:** Validated pain scales.
**Appendix S4:** Characteristics of included net‐work meta‐analysis.
**Appendix S5:** Characteristics of additional included studies—skin‐to‐skin.
**Appendix S6:** Effect of skin‐to‐skin compared to control on PIPP scores after Heel Prick.

## Data Availability

The data that support the findings of this study are available on request from the corresponding author. The data are not publicly available due to privacy or ethical restrictions.
