# Supplementary material for: Pain Management During Neonatal Blood Sampling: An Overview of Systematic Reviews
Source: Paediatr Neonatal Pain. 2026 Sep 8;8(3):e70044. doi: 10.1002/pne2.70044 (PMC13551344; doi:10.1002/pne2.70044)
Supplement: Supplementary file 1 [file PNE2-8-e70044-s001.docx]

# **Appendix S1**

Protocol for Overeview of Pain Management Strategies, Registered Prospectively on PROSPERO (CRD42023474078)

# Pain management strategies for babies during testing for neonatal hypoglycaemia: an Overview of Reviews

## Background

Neonatal hypoglycaemia is a common condition affecting up to 15% of newborn neonates (Hay et al., 2009). Severe and symptomatic neonatal hypoglycaemia can lead to poor neurodevelopmental outcomes in childhood (Adamkin, 2017). Therefore, standard clinical practice is to monitor at-risk neonates to determine need for treatment to prevent long term consequences of hypoglycaemia (Adamkin & Committee on Fetus and Newborn, 2011). This involves collecting a blood sample test for blood or plasma glucose concentration, most commonly using a heel prick (Adamkin & Committee on Fetus and Newborn, 2011). However, this is a painful procedure (Devi et al., 2023) and pain has also been suggested to have detrimental effects on neurodevelopment, especially for preterm neonates (2009). Because using painful procedures to collect blood to test for neonatal hypoglycaemia is currently unavoidable, we plan to investigate how pain can be minimised during blood sampling. We are conducting an overview of the existing systematic reviews to identify a range of effective pain management strategies. This is important as not all strategies will work for all families or in all situations.

## Method

### Searches

We will search the following electronic bibliographic databases from inception to October 2023: Ovid Medline, Embase, CINAHL Plus and the Cochrane Database of Systematic Reviews. There will be no language restrictions. The searches will be re-run just before the final analyses and further studies retrieved for inclusion. We will record the selection process in sufficient detail to complete a PRISMA flow diagram (Moher et al., 2009).

### Types of study to be included

We will include all systematic reviews that meet the Cochrane definition, “reviews of clearly formulated questions that use systemic and explicit methods to identify, select and critically appraise relevant research, and to collect and analyse data from the studies included in the reviews” (Patel et al., 2022).

Inclusion criteria for reviews:

- Source of procedural pain is blood sampling (heel prick, venipuncture, arterial puncture)

- Evidence is from randomised controlled trials (RCTs) and/or quasi-RCTs

- Search conducted within the last 10 years

- Reported search strategy

- Searched at least two databases

- Conducted risk-of-bias assessment for included studies

We will include all non-overlapping reviews. In the case of overlap, we will select the most recent systematic review.

P – neonates (up to 28 days postmenstrual age (World Health Organization, 2023) or study defined) undergoing blood sampling

I – any intervention aiming to reduce pain (pharmacological or non-pharmacological)

C – another intervention aiming to reduce pain, standard care, or no intervention

O – Primary outcome: Validated composite pain scores, measured during the procedure (pain reactivity) or after the procedure (pain response).

E.g., Neonatal Neonate Pain Scale (NIPS), Premature Neonate Pain Profile (PIPP), Neonatal Pain, Agitation and Sedation Scale (N-PASS)

Secondary outcomes:

Physiological variables - measured during the procedure (pain reactivity), after the procedure (pain response) or presented as a change from baseline.

- Heart rate
- Respiratory rate
- Oxygen saturation
- Blood pressure (systolic or diastolic)
- Cerebral oxygenation - near-infrared spectroscopy
- Brain electrical activity- electroencephalography
- Hormonal indicators from bodily fluids e.g., salivary cortisol level

Behavioural variables

- Latency of first cry
- Duration of first cry
- Duration of crying
- Percentage time crying
- Presence of facial grimace
- Percentage time grimacing
- Sleep/wake state

Adverse effects (study defined)

Parent satisfaction (study defined)

Clinician satisfaction (study defined)

Cost of intervention (study defined)

### Quality assessment

Two review authors will independently assess methodological quality of systematic reviews using the AMSTAR 2 tool. Any disagreements will be resolved by discussion and if necessary, in discussion with a third review author. Grading of Recommendations Assessment, Development and Evaluation (GRADE) approach will be used to assess the quality of evidence for the outcomes to be reported and the GradePro GDT Guideline Development Tool (https://gradepro.org/) will be used to create a ‘Summary of judgement’ table to report the quality of the key outcomes (Schünemann et al., 2013). The GRADE approach classifies the quality of a body of evidence at one of the four levels - Trivial, Small, Moderate, Large.

The outcomes to be included for GRADE assessment are:

1. Validated composite pain score
2. Adverse effects (study defined)

### Strategy for data synthesis

We will consider whether the characteristics of included reviews are sufficiently similar for meta-analysis. Meta-analyses will be undertaken using R, RevMan 5.4 or STATA using random effects modelling. We will calculate I² and χ² for each analysis and describe the percentage of variability in effect estimates that may be due to heterogeneity. The relative risks (RRs) or odds ratios (ORs) or adjusted odds ratios (aORs) with 95% confidence intervals (CIs) will be calculated for dichotomous outcomes. The mean differences (MDs) or adjusted mean differences (aMDs) with 95% CIs will be calculated for continuous outcomes. A p-value < 0.05 will denote statistical significance. A narrative summary of evidence will be provided if meta-analysis is not possible.

### Subgroup analysis

We will conduct subgroup analyses for neonates born preterm versus those born at term, and also postmenstrual age at time of assessment (<32 weeks vs 32-36weeks vs ≥37weeks).

## Discussion

This overview will summarize systematic reviews that assess the effects of interventions to minimise pain for neonates being tested for neonatal hypoglycaemia. The findings may support the development of clinical guidelines for the management of neonatal hypoglycaemia.

**Appendix S2**

**Ovid Medline**

| 1. | Pain Threshold/ or pain threshold.ti,ab,kw. |
| --- | --- |
| 2. | Pain Management/ or pain management.ti,ab,kw. |
| 3. | Pain, Procedural/ or procedural pain.ti,ab,kw. |
| 4. | Pain Measurement/ or pain measurement.ti,ab,kw. |
| 5. | Pain/ or pain.ti,kw. |
| 6. | Analgesia/ or analgesi*.ti,kw. |
| 7. | needle*.ti,kw. or Needles/ |
| 8. | (((heel adj3 prick*) or (arterial adj3 puncture*) or (artery adj3 puncture*) or (blood adj3 sampl*) or (heel adj3 lanc*) or (heel adj3 puncture*) or heel) adj3 stick*).ti,kw. |
| 9. | ((needle: adj2 (gauge: or length: or thick: or angle: or size:)) or (needle: adj2 (technique: or techinc: or aspirat: or angle: or speed: or slow: or fast: or order:))).ti,ab. or punctures/ or blood specimen collection/ or phlebotomy/ or (painf* adj2 procedur*).ti,ab. |
| 10. | (venepuncture* or venipuncture*).ti,kw. |
| 11. | 1 or 2 or 3 or 4 or 5 or 6 or 7 or 8 or 9 or 10 |
| 12. | infant/ or exp infant, newborn/ |
| 13. | ("babe" or "babes" or baby* or "babies" or "gestational age" or "gestational ages" or infant? or "infantile" or infancy or "low birth weight" or "low birth weights" or "low birthweight" or "low birthweights" or neonat* or "neo?nat*" or newborn* or "new born?" or "newly born" or "premature" or "pre?mature" or "pre-matures" or prematures or prematurity or "pre?maturity" or "preterm" or "preterms" or "pre?term" or "preemie" or "preemies" or "premies" or "premie" or "VLBW" or "VLBWI" or "VLBW-I" or "VLBWs" or "LBW" or "LBWI" or "LBWs" or "ELBW" or "ELBWI" or "ELBWs" or "NICU" or "NICUs").ti,ab,kw. |
| 14. | *Intensive Care, Neonatal/ |
| 15. | *Intensive Care Units, Neonatal/ |
| 16. | *Gestational Age/ |
| 17. | 12 or 13 or 14 or 15 or 16 |
| 18. | Systematic Review/ |
| 19. | Meta-Analysis/ |
| 20. | (systematic or structured or evidence or trials or studies).ti. and ((review or overview or look or examination or update* or summary).ti. or review.pt.) |
| 21. | ((systematic or meta) adj2 (analys* or review)).ti,kf. or ((systematic* or quantitativ* or methodologic*) adj5 (review* or overview*)).ti,ab,kf,sh. or (quantitativ$ adj5 synthesis$).ti,ab,kf,hw. |
| 22. | "meta‐analysis".pt. or ("meta‐analys*" or "meta analys*" or "metaanalys*" or "meta synth*" or "meta‐synth*" or "metasynth*").ti,ab,kf,hw. |
| 23. | (integrative research review* or research integration).tw. or scoping review?.ti,kf. or (review.ti,kf,pt. and (trials as topic or studies as topic).hw.) or (evidence adj3 review*).ti,ab,kf. |
| 24. | review.pt. and ((medline or medlars or embase or pubmed or scisearch or psychinfo or psycinfo or psychlit or psyclit or cinahl or electronic database* or bibliographic database* or computeri#ed database* or online database* or pooling or pooled or mantel haenszel or peto or dersimonian or der simonian or fixed effect or ((hand adj2 search*) or (manual* adj2 search*))).tw,hw. or (retraction of publication or retracted publication).pt.) |
| 25. | 18 or 19 or 20 or 21 or 22 or 23 or 24 |
| 26. | 11 and 17 and 25 |
| 27. | exp animals/ not humans.sh. |

**Embase**

| 1. | Pain Threshold.ti,ab,kw. or pain threshold/ |
| --- | --- |
| 2. | Pain Management.ti,ab,kw. or analgesia/ |
| 3. | procedural pain.ti,ab,kw. or procedural pain/ |
| 4. | pain management.ti,ab,kw. |
| 5. | pain/ or pain.ti,kw. |
| 6. | Neonatal Infant Pain Scale/ or neonatal infant pain scale.ti,ab,kw. |
| 7. | pain severity score/ or pain severity score.ti,ab,kw. |
| 8. | pain assessment/ or pain assessment.ti,ab,kw. |
| 9. | pain parameters/ or pain parameters.ti,ab,kw. |
| 10. | needle*.ti,kw. or needle/ |
| 11. | ((heel adj3 prick*) or (arterial adj3 puncture) or (artery adj3 puncture) or (blood adj3 sampl*) or (heel adj3 lanc*) or (heel adj3 puncture*) or (heel adj3 stick*)).ti,kw. |
| 12. | ((needle: adj2 (gauge: or length: or thick: or angle: or size:)) or (needle: adj2 (technique: or techinc: or aspirat: or angle: or speed: or slow: or fast: or order:))).ti,ab. or punctures/ or blood specimen collection/ or phlebotomy/ or (painf* adj2 procedur*).ti,ab. |
| 13. | (venepuncture* or venipuncture*).ti,kw. |
| 14. | 1 or 2 or 3 or 4 or 5 or 6 or 7 or 8 or 9 or 10 or 11 or 12 or 13 |
| 15. | newborn/ or newborn period/ |
| 16. | ("babe" or "babes" or baby* or "babies" or "gestational age" or "gestational ages" or infant* or "infantile" or infancy or "low birth weight" or "low birth weights" or "low birthweight" or "low birthweights" or neonat* or "neo?nat*" or newborn* or "new?born" or "newly born" or "premature" or "pre?mature" or "pre?matures" or prematures or prematurity or "pre?maturity" or "preterm" or "preterms" or "pre?term" or "preemie" or "preemies" or "premies" or "premie" or "VLBW" or "VLBWI" or "VLBW-I" or "VLBWs" or "LBW" or "LBWI" or "LBWs" or "ELBW" or "ELBWI" or "ELBWs" or "NICU" or "NICUs").ti,ab,kw. |
| 17. | *Intensive Care, Neonatal/ |
| 18. | *Intensive Care Units, Neonatal/ |
| 19. | *Gestational Age/ |
| 20. | 15 or 16 or 17 or 18 or 19 |
| 21. | "systematic review"/ |
| 22. | meta analysis/ |
| 23. | (systematic or structured or evidence or trials or studies).ti. and ((review or overview or look or examination or update* or summary).ti. or review.pt.) |
| 24. | ((systematic or meta) adj2 (analys* or review)).ti,kf. or ((systematic* or quantitativ* or methodologic*) adj5 (review* or overview*)).ti,ab,kf,sh. or (quantitativ$ adj5 synthesis$).ti,ab,kf,hw. |
| 25. | "meta‐analysis".pt. or ("meta‐analys*" or "meta analys*" or "metaanalys*" or "meta synth*" or "meta‐synth*" or "metasynth*").ti,ab,kf,hw. |
| 26. | (integrative research review* or research integration).tw. or scoping review?.ti,kf. or (review.ti,kf,pt. and (trials as topic or studies as topic).hw.) or (evidence adj3 review*).ti,ab,kf. |
| 27. | review.pt. and ((medline or medlars or embase or pubmed or scisearch or psychinfo or psycinfo or psychlit or psyclit or cinahl or electronic database* or bibliographic database* or computeri#ed database* or online database* or pooling or pooled or mantel haenszel or peto or dersimonian or der simonian or fixed effect or ((hand adj2 search*) or (manual* adj2 search*))).tw,hw. or (retraction of publication or retracted publication).pt.) |
| 28. | 21 or 22 or 23 or 24 or 25 or 26 or 27 |
| 29. | 14 and 20 and 28 |
| 30. | (animal/ or nonhuman/) not human/ |
| 31. | 29 not 30 |

**Cochrane Database of Systematic Reviews**

#1	(pain):ti (Word variations have been searched)

#2	analges*:ti

#3	(needle*):ti

#4	heel near/3 prick*:ti

#5	arterial near/3 punctur*:ti

#6	artery near/3 punctur*:ti

#7	blood near/3 sampl*:ti

#8	blood specimen collection:ti

#9	phlebotomy:ti

#10	heel near/3 lanc*:ti

#11	heel near/3 punctur*:ti

#12	heel near/3 stick*:ti

#13	venepuncture*:ti

#14	venipuncture*:ti

#15	#1 or #2 or #3 or #4 or #5 or #6 or #7 or #8 or #9 or #10 or #11 or #12 #13 or #14

#16	newborn:ti,ab,kw

#17	neonat*:ti,ab,kw

#18	neo-nat*:ti,ab,kw

#19	"newly born":ti,ab,kw

#20	infan*:ti,ab,kw

#21	baby:ti,ab,kw

#22	babies:ti,ab,kw

#23	babe*:ti,ab,kw

#24	gestational age*:ti,ab,kw

#25	low birth*:ti,ab,kw

#26	prematur*:ti,ab,kw

#27	pre-matur*:ti,ab,kw

#28	preterm*:ti,ab,kw

#29	pre-term*:ti,ab,kw

#30	preemie*:ti,ab,kw

#31	premie*:ti,ab,kw

#32	VLBW*:ti,ab,kw

#33	LBW*:ti,ab,kw

#34	ELBW*:ti,ab,kw

#35	NICU*:ti,ab,kw

#36	#16 or #17 or #18 or #19 or #20 or #21 or #22 or #23 or #24 or #25 or #26 or #27 or #28 or #29 or #30 or #31 #32 or #33 or #34 or #35

#37	#15 and #36

**CINAHL**

| S7 | S1 AND S2 AND S3 AND S6 |
| --- | --- |
| S6 | S4 OR S5 |
| S5 | review AND ( medline or medlars or embase or pubmed or scisearch or psychinfo or psycinfo or psychlit or psyclit or cinahl or electronic database* or bibliographic database* or computeri#ed database* or online database* or pooling or pooled or mantel haenszel or peto or dersimonian or der simonian or fixed effect or hand search* or manual* search ) |
| S4 | systematic review OR ( meta analys* or metaanalys* or meta-analys* or meta synth* or meta-synth* or metasynth* ) OR ( integrative research review* or research integration or scoping review ) |
| S3 | TI ( "babe" or "babes" or baby* or "babies" or "gestational age" or "gestational ages" or infant* or "infantile" or infancy or "low birth weight" or "low birth weights" or "low birthweight" or "low birthweights" or neonat* or "neo?nat*" or newborn* or "new?born" or "newly born" or "premature" or "pre?mature" or "pre?matures" or prematures or prematurity or "pre?maturity" or "preterm" or "preterms" or "pre?term" or "preemie" or "preemies" or "premies" or "premie" or "VLBW" or "VLBWI" or "VLBW-I" or "VLBWs" or "LBW" or "LBWI" or "LBWs" or "ELBW" or "ELBWI" or "ELBWs" or "NICU" or "NICUs" |
| S2 | ( needle OR needle* ) OR ( venepuncture or venipuncture or phlebotomy or blood test ) OR ( blood sample or blood draw or specimen or blood collection ) OR arterial puncture OR ( heel prick or heel stick or heel lance ) OR heel prick test OR heel prick screening OR heel prick sample |
| S1 | ( pain management or pain relief or pain control or pain reduction ) OR ( pain measurement or pain assessment or pain scale ) OR pain |

**Appendix S3**

**Validated Pain Scales**

| ***Tool*** | ***Components*** | ***Range*** | ***References*** |
| --- | --- | --- | --- |
| Premature Neonate Pain Profile (PIPP) | Facial actions, heart rate, oxygen saturation, gestational age, behavioural state | 0-21, higher scores indicate more pain | Gibbins et al., 2014; Stevens et al., 2014 |
| Neonatal Neonate Pain Scale (NIPS) | Facial expression, crying, breathing, and limb movements | 0-7, higher scores indicate more pain | Lawrence et al., 1993 |
| Douleur Aiguë Nouveau-né (DAN) | Facial expressions, limb movements, and vocal expression | 0-10 higher scores indicate more pain | Carbajal et al., 1997 |
| Neonatal Facial Coding System (NFCS) | Facial expressions | 0-10, higher scores indicate more pain | Grunau & Craig, 1987 |
| Neonatal Pain, Agitation and Sedation Scale (N-PASS) | Crying, behaviour/ state, facial expression, extremities/tone, and vital signs | Pain and agitation 0-10, sedation 0-10, higher scores indicate higher levels of pain/ sedation | Hillman et al., 2015 |

**References:**

Grunau RV, Craig KD. Pain expression in neonates: facial action and cry. Pain. 1987;28(3):395-410.

Carbajal R, Paupe A, Hoenn E, Lenclen R, Olivier-Martin M. DAN: une échelle comportementale d'évaluation de la douleur aiguë du nouveau-né. Archives de pédiatrie. 1997;4(7):623-8.

Lawrence J, Alcock D, McGrath P, Kay J, MacMurray SB, Dulberg C. The development of a tool to assess neonatal pain. Neonatal network: NN. 1993;12(6):59-66.

Gibbins S, Stevens BJ, Yamada J, Dionne K, Campbell-Yeo M, Lee G, et al. Validation of the premature infant pain profile-revised (PIPP-R). Early human development. 2014;90(4):189-93.

Stevens BJ, Gibbins S, Yamada J, Dionne K, Lee G, Johnston C, et al. The premature infant pain profile-revised (PIPP-R): initial validation and feasibility. The Clinical journal of pain. 2014;30(3):238-43.

Begg C, Cho M, Eastwood S, Horton R, Moher D, Olkin I, et al. Improving the quality of reporting of randomized controlled trials: the CONSORT statement. JAMA. 1996;276(8):637-9.

**Appendix S4**

Characteristics of included net-work meta-analysis

| **Author/ Year** | **Intervention** | **Date of search** | **Number of included trials (design, publication years and procedures)** | **Number of participants in included trials** | **Network characteristics** | **Risk of bias assessment** | **Certainty assessment** |
| --- | --- | --- | --- | --- | --- | --- | --- |
| Abiramalatha 2024 (1) | 51 interventions for heel-prick pain: 29 stand-alone pharmacological/non-pharmacological interventions and 22 combination interventions, including sucrose, non-nutritive sucking (NNS), breastfeeding, glucose, expressed breastmilk (EBM), skin-to-skin, facilitated tucking, swaddling, recorded music/music therapy, white noise, touch massage, topical analgesia, and intervention combinations | February 2023 | 103 randomised controlled trials (RCTs) (evaluating analgesia during heel prick in term and/or preterm infants) | 6964 infants for pain during heel prick; 6238 infants for pain at 30 seconds after heel prick | Network meta-analysis (NMA) of 51 interventions compared with no intervention/control; P-scores used for treatment ranking; inconsistency assessed using node-splitting; pairwise meta-analyses also performed | Risk of bias (ROB) 2 assessment: 36 RCTs low risk of overall bias, 39 RCTs some concerns of overall bias, 28 RCTs high risk of overall bias | GRADE used for NMA estimates. High certainty evidence for NNS+sucrose, breastfeeding, glucose, EBM, sucrose, NNS, and touch massage for pain during heel prick |
| Amuji 2026 (2) | Breastfeeding/breast milk, breastmilk odour, glucose, sucrose, local anaesthesia, NNS, mother touch, swaddling, massage, covering eyes/ears, familiar sounds (recorded maternal voice, intrauterine sounds, or music), other odours, paracetamol, prone position, and combination interventions (local anaesthesia +sucrose, sucrose+cuddling, sucrose + music therapy (Brahms' Lullaby)) | March 2023 | 37 RCTs (venipuncture, cannulation, Epicutaneo-Caval Catheter line insertion, and femoral cannulation procedures in term and preterm infants) | 3909 | Bayesian network meta-analysis including 18 interventions and a control group; direct and indirect comparisons assessed; SUCRA used for treatment ranking; inconsistency assessed using node-splitting analysis | ROB 2 assessment: 3 RCTs low risk of overall bias, 21 RCTs some concerns of overall bias, 13 RCTs high risk of overall bias | GRADE for NMA used to assess certainty of evidence for network estimates. High certainty evidence for breastfeeding and NNS; low to very low certainty for most other interventions |
| Ding 2025 (3) | Acoustic stimulation interventions: white noise, white noise plus, recorded maternal voice, recorded maternal voice plus, vocal music therapy, instrumental music therapy, music therapy plus, music therapy and recorded maternal voice plus, other non-pharmacological interventions, other pharmacological interventions, routine care, and control. | July 2023 | 28 RCTs (procedures included heel lance, venipuncture, arterial puncture, peripheral venous cannula, aspiration, and other painful procedures) | 2624 | Bayesian NMA using gemtc in R and Stata; 12 intervention categories for pain during procedures; SUCRA used for ranking; global inconsistency assessed using deviance information criterion comparison and local inconsistency by node-splitting; subgroup and sensitivity analyses performed | ROB 1 assessment: Sequence generation: 18 RCT low risk, 8 RCT unclear risk, 2 RCT high risk Allocation concealment: 7 RCT low risk, 20 RCT unclear risk, 1 RCT high risk Blinding (participants and personnel): 5 RCT low risk, 22 RCT unclear risk, 1 RCT high risk Blinding (outcome assessors): 8 RCT low risk, 18 RCT unclear risk, 2 RCT high risk Incomplete outcome data: 26 RCT low risk, 2 RCT unclear risk, 0 RCT high risk Selective reporting: 4 RCT low risk, 24 RCT unclear risk, 0 RCT high risk Other bias: 0 RCT low risk, 28 RCT unclear risk, 0 RCT high risk | Certainty assessment not reported |
| Efendi 2026 (4) | Glucose/dextrose, sucrose, and EBM compared with placebo, no treatment, or active comparators | December 2022 (database alerts through July 2024) | 42 RCTs identified; 38 RCTs included in the NMA evaluating heel-stick, venipuncture, suctioning, ROP screening, OGT insertion, IM injection, NCPAP, and other minor procedures in preterm infants | 2367 | Frequentist network meta-analysis using the netmeta package in R; direct and indirect comparisons assessed; treatment rankings generated using P-scores; heterogeneity, inconsistency, publication bias, outlier analyses, subgroup analyses, sensitivity analyses, and network meta-regression performed | ROB 2 assessment: 19 RCTs low risk of overall bias, 10 RCTs moderate risk/some concerns of overall bias, and 9 RCTs high risk of overall bias | CINeMA framework used to assess certainty of evidence across within-study bias, reporting bias, indirectness, imprecision, heterogeneity, and incoherence. Confidence ratings ranged from high to very low across outcomes; most comparisons involving expressed breast milk were supported by very low-certainty evidence |
| Hasanul Huda 2025 (5) | Non-pharmacological parental involvement interventions including kangaroo mother care (KMC), skin-to-skin contact, breastfeeding, supplemental breastfeeding, breastmilk odour, recorded maternal voice, massage, facilitated tucking, swaddling, white noise, music therapy, sucrose, expressed breastmilk (EBM), vanilla odour, mother odour, MiniMuffs, and combination interventions (KMC + placebo, KMC + sucrose, KMC + music therapy). | August 2023 | 22 RCTs (23 data points) evaluating invasive procedures in preterm infants, predominantly heel lance procedures (91.3%), with additional venepuncture and peripheral cannulation procedures | 2036 | Bayesian network meta-analysis using GeMTC; direct and indirect comparisons performed; league tables and P-scores generated; node-splitting and design-by-treatment interaction models used to assess inconsistency; leave-one-out sensitivity analyses conducted | ROB 2 assessment: 14 RCTs low overall risk of bias, 7 RCTs some concerns, 1 RCT high overall risk of bias | CiNeMA used to assess certainty of evidence. Of 210 comparisons, 44 comparisons had moderate confidence, 147 low confidence, and 19 very low confidence; no comparisons had high confidence |
| Shen 2025(6) | Multi-sensory interventions classified according to sensory modalities: tactile–auditory, tactile–kinesthetic, gustatory–auditory, tactile–visual–gustatory–auditory–olfactory, tactile–visual–gustatory–auditory–olfactory–vestibular, and tactile–visual–gustatory–auditory–olfactory–vestibular–kinesthetic. | August 2024 | 18 RCTs; 14 RCTs included in the NMA and 4 narratively reviewed due to incompatible data; procedures included venipuncture, heel-stick, ROP screening, NCPAP, gastric tube insertion, mechanical ventilation, and other painful procedures | 1408 | Frequentist NMA using Stata 18.0; six intervention nodes plus routine care; SUCRA used for treatment ranking; global inconsistency assessed using Wald test; no loops were formed, so local inconsistency was not assessed | RoB 2 assessment: 1 RCT low overall risk of bias, 1 RCT some concerns, 16 RCTs overall high risk of bias | Certainty assessment not reported |
| Tao 2025 (7) | Multisensory stimulation interventions classified by combinations of sensory modalities, including tactile–taste, tactile–auditory, tactile–tactile, tactile–kinesthetic, auditory–visual, taste–kinesthetic, tactile–taste–kinesthetic, tactile–taste–auditory, tactile–olfactory, auditory–taste, and tactile–auditory–visual–vestibular interventions. | May 2025 | 46 RCTs evaluating neonatal blood sampling procedures, including heel blood sampling and arteriovenous blood sampling | 5836 | Bayesian network meta-analysis using Stata 18.0; separate networks for arteriovenous and heel blood sampling; SUCRA used for ranking; node-splitting used to assess inconsistency; Egger’s test used for publication bias | ROB 2 assessment: 13 RCTs low overall risk of bias, 33 RCTs some concerns, 0 RCT high overall risk of bias | Certainty assessment not reported |

Abbreviations: non-nutritive sucking (NNS), expressed breastmilk (EBM); randomised controlled trials (RCTs); Network meta-analysis (NMA); Risk of bias (ROB); kangaroo mother care (KMC).

**References:**

1.	Abiramalatha T, Ramaswamy VV, Anne RP, Amuji N, Thinesh J, Venkateshwarlu V, et al. Comparative efficacy of interventions for analgesia during heel prick in newborn infants - a systematic review and network meta-analysis. Indian Pediatrics. 2024;61(9):851-75.

2.	Amuji N, Balachander B, Abiramalatha T, Kumar J, Gupta N, Anne RP, et al. Safety and efficacy of interventions for pain management during venipuncture in neonates: a systematic review and network meta-analysis. Journal of Vascular Access. 2026:11297298251407724.

3.	Ding S, Wang Q, Fu X, Huang X, Liao L, Zhang Y. Effects of acoustic stimulation on painful procedures in preterm and full-term infants: a systematic review and network meta-analysis. International Journal of Nursing Studies. 2025;165:105031.

4.	Efendi D, Kang YN, Natalia R, Milanti A, Marsubrin PMT, Kustanti CY, et al. The comparative efficacy and safety of sweet solutions to reduce preterm infants' pain levels: a systematic review and network meta-analysis. Systematic Reviews. 2026;15(1):50.

5.	Hasanul Huda M, Susanto H, Rustina Y, Hasan F, Chipojola R, Eka Putra H, et al. Comparative efficacy of non-pharmacological interventions involving parent on pain during invasive procedure among preterm baby: A systematic review and network meta-analysis. Journal of Neonatal Nursing. 2025;31(5):101717.

6.	Shen MD, Ding XD, Fu L, He HX, Chen SB, Hu YC, et al. Comparative effectiveness of multi-sensory interventions for reducing pain among premature infants: a systematic review and network meta-analysis. International Journal of Nursing Studies. 2025;161:104947.

7.	Tao C, Zhang L, Tang J, He C, Liu Q, Li Y, et al. Effect of multisensory stimuli on neonatal pain during blood sample collection: a network meta-analysis of randomized controlled trials. European Journal of Pediatrics. 2025;184(12):735.

**Appendix S5**

### Characteristics of Additional Included Studies – Skin-to-Skin

| Author/ Year | SR from | Intervention/ control | Painful procedure | Characteristics | Risk of bias | Outcomes |
| --- | --- | --- | --- | --- | --- | --- |
| Ding 2011 (8) | Wang et al. (9) | KG - 30 min of KMC before and 20 min after heel blood collection  CG - Standard care | Heel prick | N - KG: 31; CG: 31  KG: 34.52 ± 1.34 CG: 34.13 ± 1.45 W | Random sequence generation: unclear risk  Allocation concealment: unclear risk  Blinding of participants and personnel: high risk  Blinding of outcome assessment: unclear risk  Incomplete outcome data: low risk  Selective reporting: low risk  Other bias: unclear risk | NFCS at heel prick |
| Fu 2017 (10) | Wang et al. (9) | KG - 30 min of KMC before and 20 min after heel prick  CG - standard care during heel prick | Heel prick | N - KG: 150; CG: 150  KG: 34.3 ± 1.5 W CG: 34.2 ± 1.5 W | Random sequence generation: unclear risk  Allocation concealment: unclear risk  Blinding of participants and personnel: high risk  Blinding of outcome assessment: unclear risk  Incomplete outcome data: low risk  Selective reporting: low risk  Other bias: unclear risk | NFCS at heel prick |
| Liao 2020(11) | Teles et al. (12) | KG - KMC after birth no clear timing  CG - Standard care during painful procedure | Heel prick | N - KG: 39; CG: 39  KG: 40.06±0.79 W CG: 40.17±0.84 W | Random sequence generation: unclear risk  Allocation concealment: unclear risk  Blinding of participants and personnel: unclear risk  Blinding of outcome assessment: unclear risk  Incomplete outcome data: low risk  Selective reporting: low risk  Other bias: unclear risk | NIPS (unclear timing, assume after heel prick) |
| Shi 2016 (13) | Wang et al. (9) | KG - 15 min of KMC before, during and 20 min after heel blood collection  CG - Standard care during painful procedure | Heel prick | N = KG: 32; CG: 31  KG: 34.89 ± 1.65 W CG: 34.27 ± 1.49 W | Random sequence generation: unclear risk  Allocation concealment: unclear risk  Blinding of participants and personnel: high risk  Blinding of outcome assessment: unclear risk  Incomplete outcome data: unclear risk  Selective reporting: high risk  Other bias: unclear risk | DAN, timing unclear |
| Shi 2017(14) | Wang et al. (9) | KG - 90 min of KMC before and 30 min after heel blood collection  CG - Standard care during painful procedure | Heel prick | N - KG: 129; CG: 129 | Random sequence generation: low risk  Allocation concealment: unclear risk  Blinding of participants and personnel: high risk  Blinding of outcome assessment: unclear risk  Incomplete outcome data: low risk  Selective reporting: low risk  Other bias: unclear risk | NIPS after heel prick |
| Shukla 2018 (15) | Eissler et al. (16)  Sharma and Samuel (17)  Wade (18)  Wang et al. (9) | KMC at least 10 minutes before procedure/ KMC with music therapy, music therapy, standard care  All neonates received expressed breast milk | Heel prick | 200 preterm neonates (50 per group): GA: 29–36 6/7 weeks | CONSORT score  Cochrane RoB 2  Bias arising from randomisation process: low risk  Bias due to deviations from intended interventions: low risk  Bias due to missing outcome data: low risk  Bias in measurement of the outcome: low risk  Bias in selection of the reported result: low risk  Overall: low risk  Sequence generation: low risk  Allocation concealment: low risk  Blinding of participants and personnel: high risk  Blinding of outcome assessors: low risk  Incomplete outcome data: low risk  Selective outcome reporting: low risk  Other sources of bias: unclear risk  Overall: low risk  Random sequence generation: low risk  Allocation concealment: low risk  Blinding of participants and personnel: high risk  Blinding of outcome assessment: low risk  Incomplete outcome data: low risk  Selective reporting: low risk  Other bias: low risk | PIPP at 30s |
| Wang 2020 (19) | Wang et al. (9) | KG - At least 60 min KMC every day, for 14 days; 30 min of KMC before procedure  CG - Standard care | Heel prick | N - KG: 32; CG: 33 | Random sequence generation: low risk  Allocation concealment: low risk  Blinding of participants and personnel: high risk  Blinding of outcome assessment: unclear risk  Incomplete outcome data: low risk  Selective reporting: low risk  Other bias: low risk | PIPP during heel prick |

***Notes.*** RCT = randomized controlled trials; SR = systematic review; KG = kangaroo group; KMC = kangaroo mother care; CG = control group; N = sample; W = weeks; GA = gestational age; NFCS = The Neonatal Facial Coding System (20);DAN = The Douleur Aiguë Nouveau-né scale (21); NIPS = The Neonatal Neonate Pain Scale (22); PIPP = The Premature Neonate Pain Profile (23, 24): CONSORT score = score on Consolidated Standards of Reporting Trials (25).

**References:**

1.	Abiramalatha T, Ramaswamy VV, Anne RP, Amuji N, Thinesh J, Venkateshwarlu V, et al. Comparative efficacy of interventions for analgesia during heel prick in newborn infants - a systematic review and network meta-analysis. Indian Pediatrics. 2024;61(9):851-75.

2.	Amuji N, Balachander B, Abiramalatha T, Kumar J, Gupta N, Anne RP, et al. Safety and efficacy of interventions for pain management during venipuncture in neonates: a systematic review and network meta-analysis. Journal of Vascular Access. 2026:11297298251407724.

3.	Ding S, Wang Q, Fu X, Huang X, Liao L, Zhang Y. Effects of acoustic stimulation on painful procedures in preterm and full-term infants: a systematic review and network meta-analysis. International Journal of Nursing Studies. 2025;165:105031.

4.	Efendi D, Kang YN, Natalia R, Milanti A, Marsubrin PMT, Kustanti CY, et al. The comparative efficacy and safety of sweet solutions to reduce preterm infants' pain levels: a systematic review and network meta-analysis. Systematic Reviews. 2026;15(1):50.

5.	Hasanul Huda M, Susanto H, Rustina Y, Hasan F, Chipojola R, Eka Putra H, et al. Comparative efficacy of non-pharmacological interventions involving parent on pain during invasive procedure among preterm baby: A systematic review and network meta-analysis. Journal of Neonatal Nursing. 2025;31(5):101717.

6.	Shen MD, Ding XD, Fu L, He HX, Chen SB, Hu YC, et al. Comparative effectiveness of multi-sensory interventions for reducing pain among premature infants: a systematic review and network meta-analysis. International Journal of Nursing Studies. 2025;161:104947.

7.	Tao C, Zhang L, Tang J, He C, Liu Q, Li Y, et al. Effect of multisensory stimuli on neonatal pain during blood sample collection: a network meta-analysis of randomized controlled trials. European Journal of Pediatrics. 2025;184(12):735.

8.	Ding XH, Pan, W. H., Wang, S., Guo, Y., Li, S. L. . Effect of kangaroo mother care on heel prick induced pain in premature infants. Chinese General Practice. 2011;14(10):1142–4.

9.	Wang F, Zhang Q, Ni ZH, Lv HT. Effects of kangaroo care on pain relief in premature infants during painful procedures: A meta‐analysis. J Spec Pediatr Nurs. 2022;27(4):e12390.

10.	Fu DM. Analysis of the impact of kangaroo nursing on pain caused by heel blood collection in premature infants. Medical Theory & Practice. 2017;30(10):1536–60.

11.	Liao Q, Chen L, Fu S, Hu H. Effect of kangaroo mother care method on pain, growth and breastfeeding in newborns. International Journal of Clinical and Experimental Medicine. 2020;13(6):4403-8.

12.	Teles LA, Arcanjo FPN, Cardoso KM, Justino JS. Impact of skin-to-skin contact on acute procedural pain in newborns: a systematic review and meta-analysis. J Pediatr (Rio J). 2025;101(6):101442.

13.	Shi CX, Zhu, Y. Q. Clinical effect of kangaroo care on relieving pain of heel blood collection of premature infants. Journal of Clinical Medicine in Practice. 2016;20(20):212–3.

14.	Shi Min ZS, Han Ying, Dong Hongmei, Liu Li, Xue Ying. Effect of kangaroo humanistic care in pain caused by heel blood collection in preterm infants in NICU. Contemporary Nurses (First Edition). 2017;11:94-5.

15.	Shukla VV, Bansal S, Nimbalkar A, Chapla A, Phatak A, Patel D, et al. Pain Control Interventions in Preterm Neonates: A Randomized Controlled Trial. Indian Pediatrics. 2018;55(4):292-6.

16.	Eissler AB, Zwakhalen S, Stoffel L, Hahn S. Systematic review of the effectiveness of involving parents during painful interventions for their preterm infants. Journal of Obstetric, Gynecologic & Neonatal Nursing. 2022;51(1):6-15.

17.	Sharma N, Samuel AJ. A systematic review of multisensory stimulation on procedural pain among preterm neonates. Pediatr Phys Ther. 2023;35(3):286-91.

18.	Wade C, Frazer JS, Qian E, Davidson LM, Dash S, te Water Naudé A, et al. Development of locally relevant clinical guidelines for procedure-related neonatal analgesic practice in Kenya: a systematic review and meta-analysis. The Lancet Child & Adolescent Health. 2020;4(10):750-60.

19.	Wang Y. The effect of kangaroo mother care on brain function, neurobehavior and repeated procedural pain in premature infants: Southwest Medical University; 2020.

**Appendix S6**

**Effect of Skin-to-Skin Compared to Control on PIPP scores after Heel Prick**

a. At 30 s

b. At 1 min

c. At 2 min

Cong 2011 has been entered twice, as it included two sub studies, the first involving 80 minutes of skin-to-skin contact and the second 30 minutes of skin-to-skin contact.

**Effect of Skin-to-Skin Contact Compared to Control on NCFS Scores**

a. During procedure

b. After procedure

**Effect of Skin-to-Skin Contact Compared to Control on NIPS Scores**

after procedure

**Effect of Skin-to-Skin Contact Compared to Control on Heart Rate**

a. During procedure

b. After procedure
